# Benchmarking Concentration and Extraction Methods for Wastewater-Based Surveillance of Eight Human Respiratory Viruses: Implications for Rapid Application to Novel Pathogens

**DOI:** 10.1021/acs.est.4c13635

**Published:** 2025-08-29

**Authors:** Audrey Liwen Wang, Minxi Jiang, Allie Nguyen, Staci R. Kane, Monica K. Borucki, Rose S. Kantor, Kara L. Nelson

**Affiliations:** † Department of Civil and Environmental Engineering, 1438 University of California, Berkeley, California 94720, United States; ‡ Physical and Life Sciences Directorate, 4578 Lawrence Livermore National Laboratory, Livermore, California 94550, United States

**Keywords:** dPCR, virus forms, coronavirus, influenza A virus, coxsackievirus, adenovirus, PMMoV, carjivirus

## Abstract

To provide early warning and support a rapid response to a novel virus through wastewater surveillance, it would be ideal to understand in advance which concentration and extraction methods are likely to be effective for dPCR-based methods, depending on virus characteristics. In this study, we spiked raw wastewater samples with eight human respiratory viruses and processed them with four methods that concentrate and/or extract nucleic acids from both liquid and solid fractions (Promega, Nanotrap, and InnovaPrep) or only the solid fraction of wastewater (Solids). Our findings provide encouraging evidence that all four methods combined with dPCR could detect an emerging virus in wastewater, although they differed in sensitivity. The pattern of recovery efficiency for adenoviruses, coronaviruses, and influenza A viruses was consistent across methods, with Promega producing higher median recovery efficiencies, while distinct patterns were observed for coxsackieviruses. We also normalized the concentration data with two endogenous fecal indicators, PMMoV and Carjivirus (formerly crAssphage). We found that normalization could reduce method-associated differences if the indicator exhibited a recovery pattern similar to that of the target virus. These findings can guide the selection of concentration and extraction methods for wastewater monitoring based on the properties of target viruses, thus enhancing pandemic preparedness.

## Graphical abstract

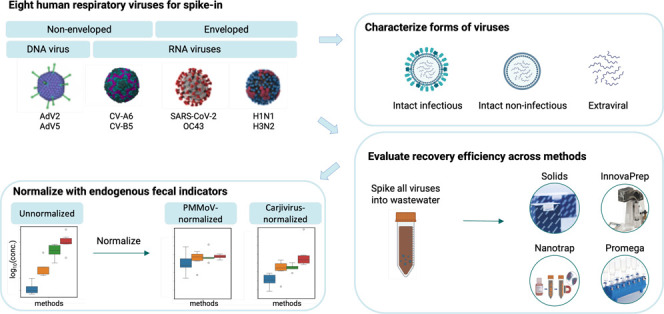

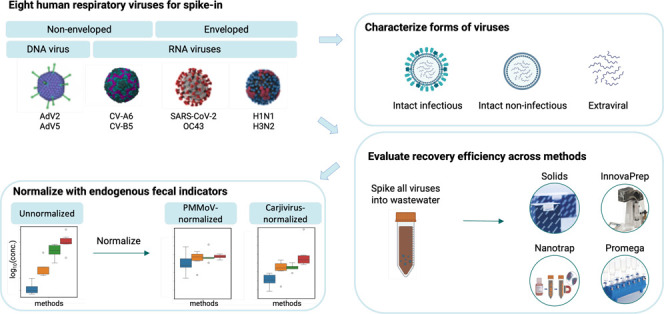

## 1 Introduction

Since the COVID-19 pandemic, there has been a rapid expansion of wastewater-based surveillance (WBS) for monitoring population-level infectious disease trends in communities.^1–4^ Many countries have launched wastewater monitoring programs for SARS-CoV-2 based on detection of nucleic acids by PCR and have added additional pathogen targets to respond to public health priorities.^5–7^ Human respiratory viruses are one of the most common causes of pandemics due to their fast transmissibility and high infectivity through respiratory droplets and aerosols.^8,9^ To prepare for future pandemics, it would be ideal to have knowledge about which methods are likely to be effective for detecting novel targets in wastewater to facilitate faster public health intervention. However, sensitive detection of viruses in wastewater remains a challenge, as different virus types, concentration and extraction methods, and wastewater characteristics can affect viral nucleic acid recoveries.

Interactions between the concentration and extraction methods and virus properties can lead to large variability in recoveries between virus types. For example, virus envelope types may influence the susceptibility to reagents as well as wastewater constituents such as detergents, solvents, and disinfectants.^10,11^ Additionally, the characteristics of surface functional groups of viruses can affect their adsorption and partitioning to particulates in wastewater.^12–14^ Adsorption in turn may affect method-specific recovery depending on the fraction(s) of a wastewater sample targeted by each method (liquid, solid, or both fractions).^15,16^ Finally, virus integrity (intact virus versus extraviral nucleic acids) and genome type (RNA versus DNA genome)^17^ could affect recovery, given that some methods extract total nucleic acids directly, while others first isolate viruses and then perform extraction.

Numerous method comparison studies highlight the challenges that result from differences in the nucleic acid recovery efficiency and the sensitivity of each method by quantitative, digital droplet, or digital PCR.^18–21^ Viruses are naturally concentrated in settled solids, and high-throughput solid-based methods have been developed.^22,23^ Meanwhile, for whole wastewater samples, viruses must be concentrated to achieve sufficient detection sensitivity. Prior to the COVID-19 pandemic, multiple concentration strategies were used, including polyethylene glycol precipitation, ultracentrifugation, skim milk flocculation, and electronegative membrane concentration.^20,24^ Several methods have since emerged that require less processing volume and processing time to reach similar sensitivity.^25–29^ The majority of method comparison studies for WBS have focused on coronaviruses and their surrogates,^18–20,28,30,31^ while a small number compared the recovery of other virus targets with diverse properties.^15,26,32,33^ To facilitate the rapid application of WBS to novel viruses, there is a need for additional method comparisons for viruses that span a larger range of physical and biological properties.

Here, we performed benchmarking of four commercial methods that have been widely adopted for wastewater surveillance since the start of the COVID-19 pandemic: (1) Promega Wizard Enviro TNA Kit (Promega) (referred to as “Promega” hereafter) for direct capture of whole wastewater, (2) AllPrep PowerViral DNA/RNA Kit (Qiagen) for solids extraction of centrifuged solids concentrate (referred to as “Solids” hereafter), (3) Nanotrap Microbiome A (Ceres Nanosciences SKU), which is an affinity-based concentration method for whole wastewater (referred to as “Nanotrap” hereafter), and (4) InnovaPrep Concentrating Pipette Select (InnovaPrep) for concentrating whole wastewater (referred to as “InnovaPrep” hereafter). We evaluated the recovery of eight respiratory viruses from four virus groups spanning a range of physical and biochemical properties: coronaviruses (SARS-CoV-2, OC43), influenza A viruses (H1N1, H3N2), adenoviruses (AdV-2, AdV-5), and coxsackieviruses (CV-A6 and CV-B5). We also compared the recovery of two commonly used endogenous fecal indicators (PMMoV and Carjivirus) across the methods. These markers have been frequently used to normalize SARS-CoV-2 concentrations in wastewater (to account differences in wastewater flows) due to their high concentrations in feces and their relatively low spatiotemporal variability.^4,34^ However, there is a lack of studies evaluating their recovery across methods and their effectiveness at normalizing the concentrations of different viruses. To interpret the results, we examined interactions among virus properties, method mechanisms, and varied wastewater characteristics. Based on the observed patterns of nucleic acid recovery across different methods and virus types, no method consistently had the highest recovery, and we provide recommendations for optimizing these techniques for the detection of other existing or emerging viruses that possess similar physical and biological properties.

## 2 Materials and Methods

Influent wastewater samples from three wastewater treatment plants (WWTPs) serving varying population sizes were collected at two time points (Figure S1a). Eight viruses were mixed and spiked into each 40 mL wastewater sample (Figure S1b). The forms of each virus stock (infectious, encapsidated but noninfectious, and extraviral nucleic acids only) were measured to provide insight into their recovery efficiencies (Figure S1b). The nucleic acid concentrations recovered by each of the four methods were quantified using digital PCR (dPCR) (Figure S1c).

### 2.1 Acquisition and Culturing of Viruses for Spiking

Two strains or species from each virus group—coronavirus (CoV), influenza A virus (IAV), adenovirus (AdV), and coxsackievirus (CV)—were selected for this study. The focus of our work on respiratory viruses stems from their public health significance in light of the COVID-19 pandemic. The selected virus targets can be transmitted via the respiratory route and are also known to be shed in feces, making them suitable for WBS. Four virus groups in our virus panel capture genetic diversity and span a range of physical and biological properties (Table 1) and are culturable in the lab. These properties make the virus panel suitable proxies for monitoring emerging respiratory threats. Because some of these viruses (e.g., CoV, AdV, and CV) also infect the gastrointestinal tract, they also provide insight into methods appropriate for enteric viruses.

**Table 1:** Physical and Biological Properties of the Spiked-In Viruses

Virus group	Strains/Species	Genome type	Virus structure	Genome size (kb)	Virion size (nm)
Coronaviruses	SARS-CoV-2, OC43	ssRNA	Enveloped	30	60–140
Influenza A viruses	H1N1, H3N2	ssRNA	Enveloped	13.5	80–120
Enteroviruses	Coxsackievirus A6, B5	ssRNA	Nonenveloped	7.6	22–30
Adenoviruses	Type 2, 5	dsDNA	Nonenveloped	35.5	90–100

For virus culturing, each virus was propagated using the specified cell lines and culture conditions based on previous protocols. Details about virus stocks and host cell information are provided in Table S1, and the propagation conditions and titer determination are described in the Supporting Information. For all viruses, the harvested cells were centrifuged at 2000*g* for 5 min to remove cell debris, and the supernatants were aliquoted into 1 mL tubes and stored at −80 °C; no further purification steps were performed. Finally, heat-inactivated SARS-CoV-2 (isolate: USA-WA1/2020, NR-52286) was obtained from Biodefense and Emerging Infections (BEI) Resources.

### 2.2 Measurement of the forms of Virus in the Stocks and Virus Cocktail Preparation

We determined the fractions of each virus stock that were (a) infectious, (b) noninfectious but encapsidated, and (c) extraviral. The infectious form was quantified by the median tissue culture infectious dose (TCID_50_/mL),^35^ while the other forms were determined based on dPCR with and without nuclease (DNase or RNase) pretreatment (Figure S2A). AdV virus stocks (DNA genome) were pretreated with DNase, and the rest of the virus stocks (RNA genome) were pretreated with RNase. The pretreatment process eliminated extraviral nucleic acids from the virus stock and ensured it was (>99%) encapsidated. All nuclease pretreatment experiments were performed in triplicate. Controls included triplicates of extracted viral nucleic acids treated with nuclease. DNase pretreatment followed a previously established protocol,^17,36^ and RNase pretreatment was also conducted according to a standard method.^17,37^ Full descriptions of both procedures can be found in Supporting Information Method B.

Virus stocks were extracted using the solids extraction protocol of the AllPrep PowerViral DNA/RNA Kit (Qiagen) with a modification of adding carrier RNA (Applied Biosystems) to improve binding of viral RNA to the membrane and limit possible RNA degradation.^38^ Briefly, 200 μL of virus stock was added to PowerBead tubes, followed by the addition of PM1 and a β-mercaptoethanol solution. The tubes were vortexed for 10 min and then centrifuged at 13,000 g for 1 min at room temperature. The supernatant was collected, and 6 μL of carrier RNA solution (1 μg/μL) was added to achieve a final concentration of approximately 0.01 μg/μL. The solution was incubated at room temperature for 5 min. Inhibitor Removal Solution (IRS) was then added, and the remaining steps of the protocol were followed as instructed. Finally, all virus concentrations were quantified with dPCR.

Virus cocktails were prepared 1 week before the methods comparison experiment began. Based on gene copy concentrations measured by dPCR, we prepared a single large-volume virus cocktail by combining eight virus stocks to achieve approximately 10^6^ gene copies of each virus. Aliquots of the virus cocktail were stored at −80 °C until use. For each batch experiment, one aliquot was thawed, ensuring that all batches used a virus cocktail that underwent one freeze–thaw cycle.

### 2.3 Wastewater Collection, Virus Cocktail Spike-In, and Incubation

24 h composite samples of influent wastewater were collected from three WWTPs in the greater San Francisco Bay Area. The sampling locations were the East Bay Municipal Utility District (EBMUD), West County Wastewater District (WCWD), and Sausalito Marin City Sanitary District (SMCSD), which correspond to large (∼700,000), medium (∼70,000), and small (∼18,000) populations, respectively. For each facility, samples were collected on two dates, approximately one month apart, which serve as replicates and help ensure that the wastewater composition is representative of each site: EBMUD samples on 7/26/2023 and 8/30/2023, WCWD samples on 7/31/2023 and 9/5/2023, and SMCSD samples on 8/2/2023 and 9/19/2023. This resulted in a total of six batches of samples. No rainfall occurred during the months of sample collection. All samples were transported to the laboratory on ice. Wastewater metadata including flow rate, biological oxygen demand (BOD_5_), total suspended solids (TSS), and pH were provided by wastewater agencies (Table S2).

Due to the time for wastewater sample collection being limited by the agencies (e.g., samples available in the afternoon/evening), it was not feasible to complete all experimental steps in one single day. Therefore, we designed our experiment as a two-day protocol, which was performed for each batch of wastewater. There were six batches in total, corresponding to two time points at each of the three facilities.

The two-day protocol is briefly described as follows: on the first day, once the wastewater sample was transported to the lab, it was homogenized and distributed into 15 aliquots of 40 mL each. Among the 15 aliquots, 12 (four methods × three biological replicates) were spiked with a virus cocktail, and three (Promega method × three biological replicates) remained unspiked to quantify endogenous viruses in wastewater. Then, all 15 wastewater aliquots were rotated at 20 rpm for 3 h at room temperature using Multi-Purpose Tube Rotators (Fisher Scientific) to reach equilibrium partitioning of viruses to solids.^45^ Following incubation, we stored both the wastewater samples and the remaining virus cocktail at 4 °C overnight. Storing the remaining virus cocktail overnight with the wastewater samples was used to account for virus degradation during refrigeration. On the second day, we re-equilibrated all samples on the rotator and then performed all four methods in parallel (outlined in Method [Sec sec2.4]), alongside the extraction of the virus cocktail (outlined in Method [Sec sec2.2]).

### 2.4 Concentration and Extraction Methods

Four concentration and extraction methods that employed different mechanisms were performed in parallel (Table 2). In this study, “concentration” means that influent wastewater is reduced to a smaller volume before nucleic acid extraction (cell lysis, nucleic acid binding to columns, washing of impurities, and elution of nucleic acids from the columns) occurs. We selected these four methods based on their widespread use since the pandemic, distinct working mechanisms, and because they target different wastewater fractions (solid vs whole), altogether allowing us to have different perspectives for result interpretation. To standardize the four protocols for comparison, the starting material was 40 mL of whole wastewater for all methods, and each method resulted in 100 μL of purified nucleic acids. The purified nucleic acids were aliquoted and stored at −20 °C and went through one freeze–thaw before dPCR quantification.

**Table 2:** Key Steps for Each of the Four Methods^a^

	**Step 1:** preprocessing (to avoid column/filter clogging)	**Step 2:** concentration	**Supernatant** (Promega) or **Concentrate** (other methods)	**Step 3:** extraction	**Effective volume (mL)**	**End product**
**1. Promega**	Solids removal by centrifugation at 3000*g* after protease addition	N.A	Around 40 mL of liquid supernatant	Promega liquid extraction and purification (whole supernatant processed)	∼40 mL	100 μL purified nucleic acids^b^
**2. Nanotrap**	N.A	Whole WW concentration by magnetic Nanotrap microbiome A particles	Nanotrap microbiome A particles concentrate with attached viruses	PowerViral liquid extraction (whole concentrate processed)	40 mL	100 μL purified nucleic acids^c^
**3. InnovaPrep**	Solids removal by centrifugation at 7000*g* after Tween 20 addition	Liquid concentration by ultrafilter concentrating pipette tip	170–720 pL of eluted liquid concentrate	PowerViral liquid extraction (200 pL subsample of concentrate)	11.1–40 mL	100 μL purified nucleic acids^d^
**4. Solids**	N.A	Solids concentration by centrifugation at 20,000*g*	0.22–0.82 g of pelleted solid concentrate	0.25 g PowerViral solids extraction (0.25 g subsample of concentrate)	12.9–40 mL	100 μL purified nucleic acids^e^

a The starting material for all four methods was 40 mL of raw wastewater spiked with the virus cocktail.

b Viruses that were originally present in the supernatant and that were released from solids by protease addition (remained in the supernatant after removing solids at 3000*g*).

c Viruses in the whole WW sample that attached to the Nanotrap Microbiome A Particles.

d Viruses that were originally present in the supernatant and that were released from solids by Tween 20 addition (remained in the supernatant after removing solids at 7000*g*).

e Viruses that were attached to the solids that were pelleted at 20,000*g*.

The AllPrep PowerViral DNA/RNA Kit (Qiagen) was used as the downstream extraction method for all three concentration-based methods (Nanotrap, InnovaPrep, and Solids) to ensure a consistent basis for comparing recovery efficiencies across the methods. The AllPrep PowerViral DNA/RNA Kit has the advantage of being able to extract total nucleic acids from both solid^39^ and liquid^40^ samples. In addition, it is one of the suggested extraction protocols for different concentration methods. Accordingly, we used the AllPrep PowerViral DNA/RNA Kit’s liquid extraction protocol for both the Nanotrap and InnovaPrep methods’ liquid concentrate and its solid extraction protocol for the pellet solids concentrate from the Solids method (Table 2). The Promega method is a direct capture approach that does not include a concentration step (Table 2).

In our manuscript, we refer to three methods—Promega, Nanotrap, and InnovaPrep—as “whole wastewater” methods, as they aim to recover viruses from both the liquid and solid fractions of a 40 mL wastewater sample. Although Promega and InnovaPrep include a solids removal step, both methods incorporate reagents beforehand to release viruses from solids (e.g., protease in Promega and Tween 20 in InnovaPrep). Therefore, we classified them as methods that recover viruses from both fractions. On the other hand, the Solids method aims to recover viruses from only the solid fraction.

#### 2.4.1 Promega

Promega extraction on whole wastewater was performed according to the manufacturer’s instructions (A2991 protocol). Briefly, 0.5 mL of protease solution was added to the 40 mL wastewater sample and was mixed by inversion. The sample was incubated for 30 min at ambient temperature, and then large particles were removed via centrifugation at 3000 x *g* for 10 min. Binding buffers and isopropyl alcohol were added to the resulting supernatant, and the mixture was passed through a PureYield Binding Column. Following two wash steps, the total nucleic acids were eluted in 1 mL of nuclease-free water and further purified on a PureYield Minicolumn to produce a final volume of 100 μL of purified nucleic acids.

#### 2.4.2 Solids

This method was performed according to the AllPrep PowerViral DNA/RNA Kit’s solids extraction protocol, which started with centrifuging a 40 mL wastewater sample at 20,000*g* for 10 min to obtain solids concentrate. The total weights of the pelleted solid concentrate (ranging from 0.22 to 0.82 g of wet weight) were recorded (Table S3). Then, subsampling of 0.22–0.25 g solids concentrate (resulting in an effective volume of 12.9–40 mL) was loaded in the Powerbead tubes with the addition of PM1 and Beta-mercaptoethanol solution for 10 min vortexing. The resulting supernatant was loaded onto the MB Spin Column following two washes and final elution, yielding 100 μL purified nucleic acids. We accounted for the subsampling of solids concentrate in our back-calculation of recovered viral concentration (Supporting Information Method C—eq S2).

#### 2.4.3 Nanotrap

Nanotrap was performed according to the protocol titled “Nanotrap Microbiome A: 35 mL Manual Protocol with the AllPrep PowerViral DNA/RNA Mini Kit (Ceres Nanosciences SKU)” (protocol: APP-091 December 2022). The starting wastewater volume was increased from 35 mL (original protocol) to 40 mL, and all other reagents were correspondingly scaled. Briefly, 115 μL of Nanotrap Enhancement Reagent 2 and 600 μL of Nanotrap Microbiome A Particles were sequentially added to the whole wastewater sample and mixed by inversion. The sample was incubated for 30 min at ambient temperature. Then, a magnetic rack was applied to separate the Nanotrap particles from the solution. The supernatant was discarded, and the Nanotrap particles were resuspended in 1 mL of nuclease-free water. A magnetic rack was further applied to separate out the Nanotrap particles, and the supernatant was discarded. Particles were resuspended in 600 μL of PM1 and Beta-mercaptoethanol solution, followed by 95 °C heating for 10 min to lyse the cells. Subsequent nucleic acid extraction steps followed the protocol. Note that this experiment was performed in 2023, using protocol APP-091 (December 2022). The current version is February 20, 2024, and includes several modified steps: Nanotrap beads are resuspended in EB2 instead of nuclease-free water, the addition of Beta-ME has been removed, and the incubation temperature has been changed to 70 °C instead of 95 °C. These differences may affect comparisons to other laboratories.

#### 2.4.4 InnovaPrep

InnovaPrep began by adding 5% of Tween 20 (Thermo Scientific) to the 40 mL wastewater sample and mixed by inversion to release virus particles adsorbed to solids.^41^ The sample was then centrifuged at 7000*g* for 10 min, and the supernatant was collected and passed through an Ultrafilter Concentrating Pipette Tip (CC08004 Unirradiated, InnovaPrep) to concentrate the viruses. The virus concentrate was eluted by elution fluid Tris (InnovaPrep) into a viral liquid concentrate (ranging from 170 to 720 μL, Table S3). Then, subsampling of the 170–200 μL liquid concentrate (resulting in an effective volume of 11.1–40 mL) was extracted according to the Qiagen AllPrep PowerViral DNA/RNA Kit’s liquid extraction protocol, yielding 100 μL of purified nucleic acids. We accounted for the subsampling of liquid concentrate in our back-calculation of recovered viral concentration (Supporting Information Method C—eq S2).

### 2.5 Quantification of Extracts by dPCR and Inhibition Testing

All virus targets were quantified by using the QIAcuity Four Platform Digital PCR System (Qiagen). The virus assays were designed using PriMux^42^ and Primer3Plus^43^ (Table S4, assay design details in Supporting Information Method D). All materials and conditions were summarized in Table S5. The reaction mixtures for RNA viruses (Table S5) were prepared using the QIAcuity OneStep Advanced Probe Kit (Qiagen), while the QIAcuity Probe Master Mix (Qiagen) was used for DNA viruses. The virus assays were duplexed for the same virus group. The reaction mixtures were loaded to either 24-well/96-well 8.5k plates for quantifying spiked viruses and endogenous fecal indicators or 24-well 26k plates for quantifying other endogenous background viruses. Priming and imaging used default settings, and thermal cycling conditions are reported in Table S5. Partition volume is 0.2 nL. Valid partition counts ranged from 7060 to 8293 per well for 8.5k plates and 20,231 to 25,492 per well for 26k plates (one NTC had only 11,638 valid partitions for a 26k plate, but no positive partition shown, so we excluded that sample). The operational limit of detection (LOD) is 0.312 cps/μL for the 8.5k plate and 0.078 cps/μL for the 26k plate at 95% Confidence Interval (CI) (details on the QC and determination of the instrumental LOD/LOQ are provided in Table S6 and Supporting Information Methods E). Note that concentrations measured below the 95% LOD should not be instinctively classified as nondetections; they might be detected but at a likelihood less than 95%. The positive control was linearized plasmid DNA (SARS-CoV-2) or gBlock standards from Integrated DNA Technologies, and the negative control was nuclease-free water. All positive controls showed clear separation between positive and negative partitions, while negative controls showed zero positive partition (Figure S3A). Fluorescence plots for each virus assay from the wastewater results are shown in Figure S3B. Data were analyzed using the QIAcuity Suite Software V1.1.3 (Qiagen) with automated settings for threshold and baseline, followed by manual inspection and adjustment. To test for inhibition, each extracted sample was run at 1:5 dilution with the aim to reduce the concentration of potential inhibitors. The Environmental Microbiology Minimum Information Checklist^44^ is provided in Table S7.

### 2.6 Data Analysis

All data analysis was performed in Python (v3.10.12) using package scipy.stats (v1.11.4) and scikit-posthocs (v.0.9.0), and statistical significance was determined at a 95% confidence interval (*p* < 0.05). Kruskal–Wallis H-test was employed to compare differences among concentration and extraction methods within the same virus type. Dunn’s test with Bonferroni correction was used as a posthoc test following a significant result in a Kruskal–Wallis test. All results of statistical testing (*p*-values) are reported in Table S8. Equations for calculating virus concentration in purified total nucleic acids (μL) and recovery efficiency (%) are provided in Supporting Information Method C.

## 3 Results

Eight human viruses (Table 1) were spiked into raw wastewater samples from three treatment facilities at two time points (Figure S1). Samples were homogenized and incubated to reach equilibrium and then processed in parallel by four virus concentration and/or extraction methods.

### 3.1 Recovery Efficiency Varied by Method and by Virus

Recovery efficiencies were calculated for each method and each spiked-in virus (eq S3 in Supporting Information Method C; Figure 1A) from the dPCR measurements. The initial virus concentrations measured in the concentration of the pure virus cocktail after 4 °C refrigeration overnight varied across the six batches and the eight viruses (Figure S4A). Because of this variation, it is misleading to infer recovery efficiency directly from the recovered virus concentrations (Figure S4C). Thus, the recovery efficiency was calculated for each sample batch using the measured initial concentrations for each virus (Supporting Information Methods C. Equations). The endogenous wastewater concentrations of the spike-in viruses were confirmed to be >100-fold lower than the spike-in levels (Figure S4B). While we measured only endogenous concentrations using the Promega method, this was a conservative approach because this method had the highest recovery efficiency for SARS-CoV-2, which was the virus present in the highest endogenous concentrations. Note that several measurements of recovered spiked virus quantities showed nondetections, including H1N1 by InnovaPrep, Solids, and Nanotrap and H3N2 by Solids and Nanotrap (Figure S4C), which is likely due to their lower starting concentrations caused by 4 °C refrigeration (Figure S4A). For endogenous fecal indicator viruses, instead of recovery efficiency, the amount of each virus recovered was directly compared because they were endogenous, and the starting concentrations were not known (Figure 1B).

**Figure 1:**
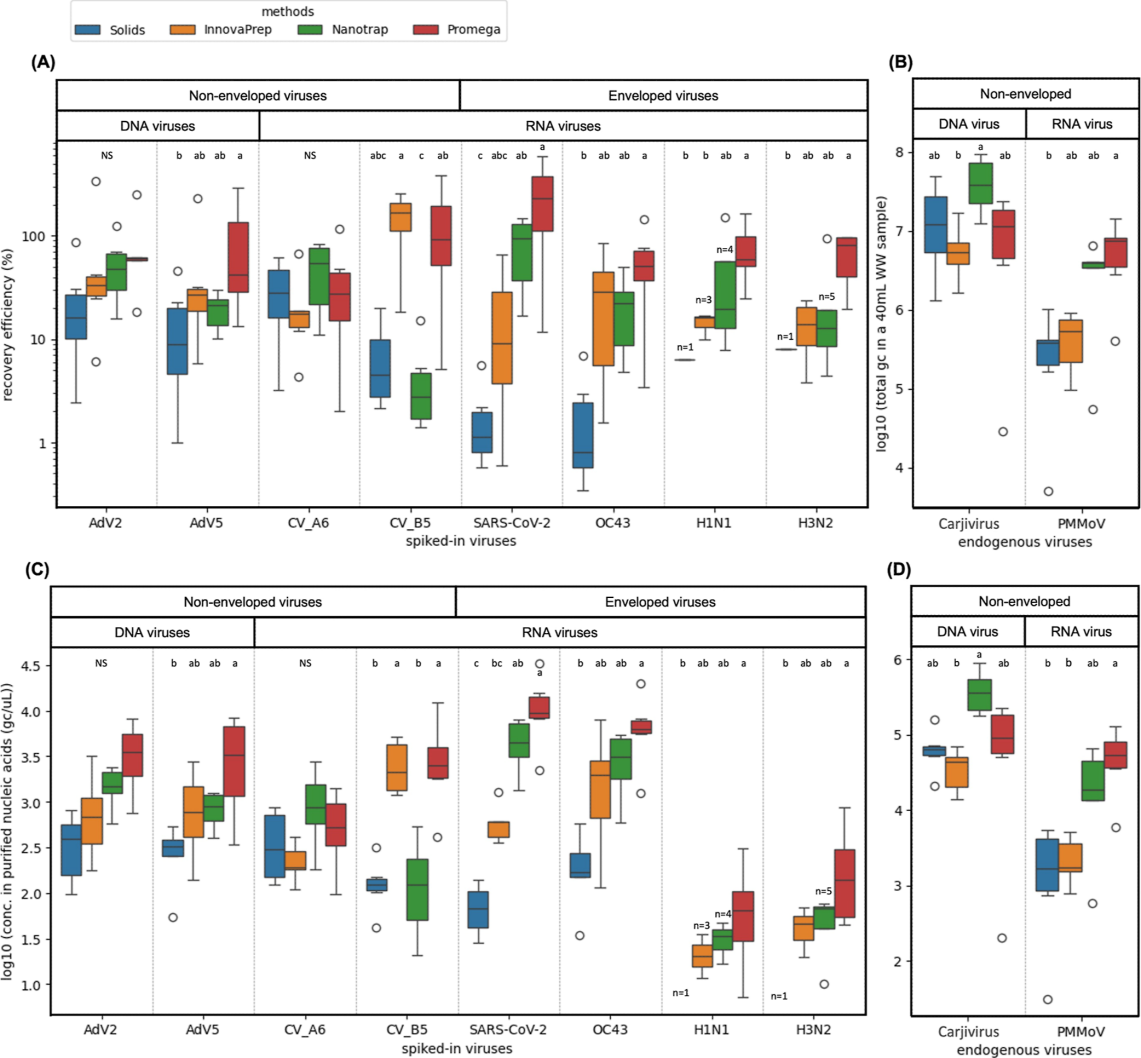
(A) Recovery efficiency (%) of the spiked-in viruses across the methods. (B) Total gene copies of endogenous fecal indicators in a 40 mL sample across methods. Concentrations of the (C) spiked-in viruses and (D) endogenous fecal indicators in purified nucleic acids (gc/μL) across four methods imply that the limit of detection (LOD) varied for each method and virus. For all figures, boxes and whiskers indicate the interquartile range (IQR) and minimum/maximum values within 1.5 times the IQR, respectively, across all samples (*n* = 6 for two time points and three wastewater sources; three biological replicates from each wastewater were first averaged by the geometric mean). The total number of samples (*n*) is shown when less than 6, due to concentrations not detected on dPCR. Outliers are displayed as individual points. Significance letters are shown based on the results of the posthoc Dunn’s test for pairwise comparisons of methods within each virus type. Any methods sharing the same letter are not significantly different from each other. NS = no significant differences.

Mean recovery efficiencies ranged from approximately 0.34% (OC43 extracted by Solids) to around 400% (SARS-CoV-2 extracted by Promega) (Figure 1A). The recovery efficiencies that exceed 100% highlight one of the challenges in accurately quantifying virus recovery, which is further discussed in Discussion [Sec sec4.2]. Among the enveloped viruses, strains from the same virus group displayed a similar recovery pattern across the four methods, and Promega exhibited higher recovery than Solids (*p*_dunn_ < 0.05, Table S8). Among the nonenveloped viruses, the two adenoviruses showed similar recovery trends across different methods, but the two coxsackieviruses differed. Recovery of CV-A6 was not significantly different by any method, whereas CV-B5 showed higher recovery with Promega and InnovaPrep and lower recovery with Nanotrap. Lastly, the pattern of recovery for endogenous PMMoV across methods was similar to that of adenoviruses, coronaviruses, and influenza A viruses (Figure 1B; Promega > Solids, *p*_dunn_ = 0.013, Table S8). Meanwhile for endogenous Carjivirus, all methods performed similarly, though InnovaPrep appeared slightly less efficient (statistically significant only between InnovaPrep and Nanotrap).

### 3.2 Sensitivity and Inhibition Varied across Methods

To understand differences in method sensitivity from a practical perspective, concentrations of each virus genome in the purified nucleic acids (the input material for dPCR) were calculated for all methods (eq S1 in Supporting Information Method C; Figure 1C,D). The Solids and InnovaPrep methods were characterized by low sensitivity relative to other methods for most targets (Figure 1B); the exception was CV-B5, which was well-recovered by InnovaPrep. Limitations of the extraction kit prevented extraction of the entire sample using these methods. The average effective volume processed was 27 mL for InnovaPrep and 30 mL for Solids, compared to 40 mL for Promega and Nanotrap (Table S3). Additionally, we observed variable levels of inhibition across different wastewater sources, dependent on the dPCR assay (Figure S5). Where inhibition was observed, it was higher with Promega compared with the other methods. Across the three treatment plants, SMCSD showed higher inhibition with this method than at the other locations. Among all spiked-in viruses, both adenoviruses showed no inhibition across all three treatment plants. Since adenoviruses are the only DNA viruses, the inhibition likely occurs during the reverse transcription step for other RNA viruses. Note that we did not correct for inhibition in any of our calculations (e.g., Figure 1).

### 3.3 Forms of Viruses for Each Virus Stock Differed

Previous research found that the predominant virus form measured in wastewater was encapsidated virus.^17^ We were concerned that the forms could vary across each virus stock and could affect the performance of the different methods. Therefore, we determined the fractions of each viral stock that were (a) intact infectious, (b) intact noninfectious (protected from digestion by nucleases), and (c) extraviral (nonintact and noninfectious free nucleic acids) (Figure 2). The infectious form was quantified by TCID_50_, while the other forms were determined based on dPCR with and without nuclease pretreatment (Figure S2). Both coxsackieviruses were primarily intact but noninfectious (CV-A6 = 75.61.45% and CV-B5 = 77.81.1%). Adenovirus Type-2 and Type-5 contained 58.51.51% and 45.81.24% extraviral nucleic acids, respectively. Interestingly, all of the nuclease-protected AdV5 was infectious (54.31.14%), whereas only around 5% of the total AdV2 was infectious. Influenza A viruses were primarily extraviral (both strains around 75%), while nearly all of the OC43 stock consisted of nuclease-protected but noninfectious forms (87.71.13%). Lastly, the heat-inactivated SARS-CoV-2 was primarily extraviral (83.81.4%), while the TCID_50_ was certified to be zero by the vendor. We note that virus forms may have changed during incubation in wastewater, but we did not attempt to account for potential changes.

**Figure 2:**
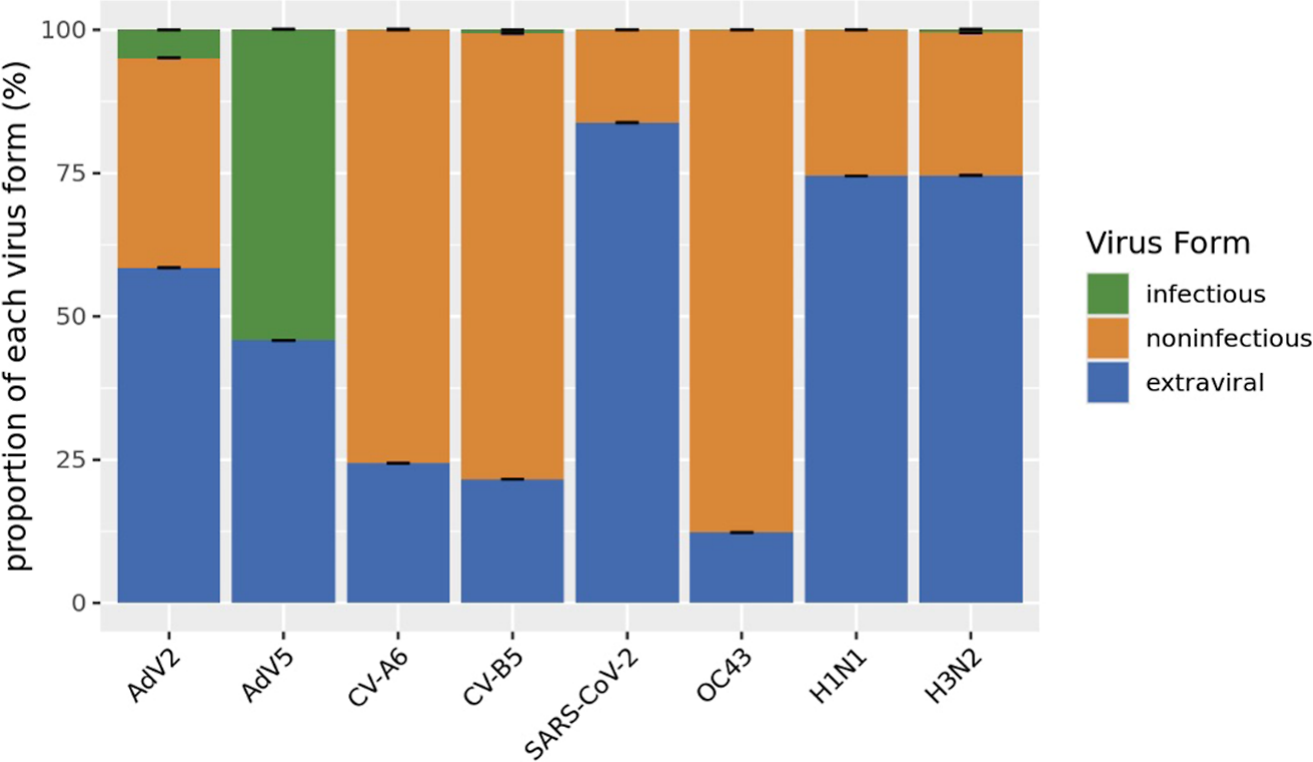
Virus stocks were characterized to determine intact infectious (green), intact noninfectious (orange), and extraviral (blue) fractions. To determine these fractions, three measurements were taken: TCID_50_ (intact infectious), intact nucleic acids after DNase/RNase treatment (= encapsidated form = intact infectious plus intact noninfectious), and total nucleic acids (= all three forms). The intact noninfectious form was the difference between the encapsidated form and TCID_50_. The extraviral form was obtained from subtracting total nucleic acids with the encapsidated form. TCID_50_ was not determined for SARS-CoV-2, which was purchased as an inactivated stock. The standard deviation for each proportion was calculated by using error propagation.

### 3.4 Solids Partitioning Estimates Differed by Method

For compatibility with other methods comparison studies,^12,22,23,45^ we calculated virus concentrations on a per-mass basis (gc/gTSS for the Solids method and gc/mL wastewater for the other methods). When compared on a per mass basis, all targets were higher in Solids samples than in whole wastewater samples extracted by InnovaPrep, Promega, and Nanotrap (Figure S6). To determine which viruses were most enriched in the solids fraction of wastewater on a mass equivalent basis, we calculated ratios of mass-equivalent gene concentration of Solids method (gc/gTSS) to the whole wastewater method (gc/mL WW) (Table 3). In this context, it is assumed that 1 mL of water has a mass of approximately 1 g. Note that these ratios are not distribution coefficients^45^ because all three of the whole wastewater methods were designed to capture viruses from both the liquid and the solids fractions of the sample. As expected, the ratios depended on the whole wastewater methods, as has been shown previously,^23^ with lower ratios found for Promega. If different Solids methods were used, the ratio could also differ depending on the Solids method. Ratios also varied up to 100-fold between viruses; e.g., for Solids/InnovaPrep, the ratio was 4550 mL/g for CV-A6 and 104 mL/g for Influenza A H1N1. Surprisingly, most ratios were lower for enveloped viruses, which were expected to have higher solids association than the nonenveloped viruses. We note that there is low confidence in the IAV concentrations measured by the Solids method because many samples had no virus detected (Figure 1C).

**Table 3:** Mass-Equivalent Gene Concentration Ratios (mL/g) of Solids to InnovaPrep, Nanotrap, and Promega for Spike-In Viruses and Endogenous Fecal Indicators^a^

virus targets		InnovaPrep (mL/g)	Nanotrap (mL/g)	Promega (mL/g)
Spiked-in viruses	AdV2	1200 ± 1.89	1250 ± 1.81	598 ± 2.00
	AdV5	864 ± 1.99	1740 ± 1.89	270 ± 2.26
	CV-A6	4550 ± 1.67	1570 ± 1.84	2560 ± 1.85
	CV-B5	154 ± 2.11	4800 ± 2.21	201 ± 2.15
	OC43	269 ± 2.95	484 ± 2.56	179 ± 2.58
	SARS-CoV-2	373 ± 2.44	87.2 ± 1.90	30.7 ± 1.98
	H1N1	104 ± 1.60	139 ± 1.43	22.0 ± 1.76
	H3N2	244 ± 1.71	357 ± 1.59	44.1 ± 1.68
Endogenous fecal indicators	PMMoV	1870 ± 2.27	259 ± 3.02	125 ± 2.43
	Carjivirus	6670 ± 1.88	1000 ± 1.88	3680 ± 3.63

a Each value shown is the geometric mean of six samples (*n* = 6 for two time points and three wastewater sources; three biological replicates from each wastewater were first averaged by the geometric mean as well), except for several IAV samples that showed non-detection. The geometric standard deviation was calculated by using error propagation. The ratios were calculated on a mass-equivalent basis, and the values were rounded to three significant figures.

### 3.5 Normalization Using Fecal Indicators

Endogenous fecal indicators are commonly used to normalize the fecal strength of wastewater (e.g., accounting for dilution by infiltrating groundwater or stormwater). In addition, they can potentially be used to account for differences in recovery efficiency between methods so that wastewater concentrations can be compared across data sets generated with different methods.^19^ The results from normalizing the recovered viral gene copies of the spiked-in viruses (Figure S4C) using PMMoV and Carjivirus are shown in Figure S7A.

We found that normalizing reduced differences between methods when the endogenous fecal indicator exhibited a recovery pattern similar to that of the target virus across the methods. However, neither indicator was appropriate for all virus targets, and in some cases, normalization increased the differences in recovery between methods.

PMMoV eliminated the statistically significant differences observed between methods for both coronaviruses, both influenza A viruses and AdV5. However, statistically significant differences between methods were introduced for AdV2 and CV-A6 (Figure S7B). In contrast, there were no statistically significant differences after normalizing CV-A6 by Carjivirus, similar to the unnormalized data. For CV-B5, its recovery pattern did not resemble that of either PMMoV or Carjivirus, and normalization with either virus did not remove the statistical difference across methods, although the differences between the concentrations measured by Solids and Promega were reduced by PMMoV normalization.

## 4 Discussion

### 4.1 Interactions between Virus Characteristics and the Underlying Mechanism of Each Method May Affect Recovery

To understand the observed recovery efficiencies, we explore how the capsid properties and forms of the different viruses may have interacted with the specific mechanisms employed in these four methods. We emphasize that the goal of this study was not to identify the “best” method, rather, it was to gain insight into which methods perform better or worse depending on the virus targets and their properties.

#### 4.1.1 Promega

The high recoveries by Promega reported previously for SARS-CoV-2 were also observed for all viruses studied here.^25,29^ Promega is a whole wastewater method that is designed to recover viruses from both the liquid and solid fractions of a 40 mL wastewater sample. The protease treatment before the 3,000*g* solids removal step seems to be effective for the lysis of viral protein capsids and release of nucleic acids, as has been previously demonstrated with other proteases.^46,47^ We reasoned that by targeting whole wastewater and having a high effective volume close to 40 mL, Promega appears to be less biased toward different viral forms and virus partitioning to solids, hence leading to higher recoveries. Additionally, the direct capture approach eliminates the need for a prior concentration step, which may reduce losses compared to multiple-step concentration–extraction approaches.^21^ Notably, Promega also had higher levels of dPCR inhibition for RT-dPCR assays (Figure S5), which may also explain why the recovery of coxsackieviruses was not higher. The inhibition testing also supports that coxsackieviruses have high inhibition for the Promega method (Figure S5). Additional inhibitor removal steps may improve the results for this method.

#### 4.1.2 Nanotrap

This method generally performed better than InnovaPrep and Solids, which is likely attributed to its high effective volume (40 mL; Table 2). Nanotrap particles are magnetic hydrogel particles coupled with high-affinity baits that bind to a broad range of proteins.^48,49^ The composition of the baits determines the targets.^50–52^ For instance, Nanotrap particles containing the affinity dye Cibacron Blue were most effective at capturing rift valley fever virus (RVFV) and human immunodeficiency virus type 1 (HIV-1),^50^ while particles with red baits were more effective for concentrating influenza A viruses.^52^ The low recovery of CV-B5 by Nanotrap in our study was an exception to the generally good recovery for the other viruses; it is possible that the mixture of baits in the Microbiome A reagent had a lower affinity for this virus due to its capsid structure. Although both CV-A6 and CV-B5 possess the four structural proteins VP1–VP4 that form the viral capsid, variations in the surface-exposed loop regions of these proteins can create distinct capsid properties.^53,54^ For example, several studies have shown that CV-B5 exhibits greater resistance to chemicals for degradation, likely due to a more stable and well-shielded capsid surface.^55,56^ The differences in capsid surface features may influence how Nanotrap affinity baits interact with each virus, potentially affecting capture efficiency and specificity. Given that the recovery efficiencies of CV-A6 and CV-B5 differed by around 20-fold, caution should be exercised in extrapolating the performance of Nanotrap to new viruses, even those that are closely related. While these differences are not inconsistent with previous findings about other differences between A6 and B5, because of the high variability, more research is needed to understand whether the different performance of Nanotrap is consistently observed. A previous spike-in study comparing Nanotrap with PEG precipitation for SARS-CoV-2, influenza, measles viruses, and norovirus also found that recoveries by Nanotrap were dependent on virus types.^33^ Another SARS-CoV-2 spike-in study reported significantly higher recovery using Nanotrap Microbiome A particles compared to traditional membrane filtration and skim milk workflows.^28^ Note that Beta-ME and 95 °C incubation were applied according to the latest available version of the Nanotrap protocol at the time of the experiment. Different versions of the Nanotrap protocols may affect the recovery of some viruses. Additionally, we did not remove solids via centrifugation prior to using the Nanotrap beads, whereas other laboratories may, and our results may not reflect the recoveries achieved with a solid removal step.

#### 4.1.3 InnovaPrep

InnovaPrep concentrates viruses via size exclusion,^57^ and we selected the Concentrating Pipette Tips characterized as ultrafiltration (specific pore size not reported for product CC08004). Despite the possibility that the pores might not capture extraviral nucleic acids as effectively as intact viruses, we did not observe a trend between lower recovery efficiency and the percentage of extraviral nucleic acids; a more controlled study would be needed to isolate this factor. InnovaPrep yielded higher recovery efficiency for CV-B5 than for other viruses, including CV-A6 (Figure 1A). We speculated that possible reasons for the higher recovery efficiency of CV-B5 relative to other viruses include that it had lower adsorption to wastewater particles, was more effectively released from wastewater particles by the addition of Tween 20 prior to solids removal, or was more stable in the wastewater and in the presence of Tween 20. Our findings are consistent with a recent study that found CV-B5 was not effectively removed by activated sludge treatment via sorption to biological flocs or degradation, compared to several other viruses that had higher association with biological flocs.^14^ More generally, enteroviruses have been shown to have widely different susceptibility to chemicals (such as chlorine^56^) due to differences in their capsid structures, reinforcing that different behaviors can be observed among closely related viruses. Two previous studies, which involved spike-in of SARS-CoV-2, influenza A and B and murine hepatitis virus (MHV; a coronavirus surrogate), have reported lower recovery by InnovaPrep compared to centrifugal ultrafiltration.^31,32^ Nonetheless, Forés et al. still recommended InnovaPrep due to its high concentration factor and low limit of detection.^31^

#### 4.1.4 Solids

The low recovery efficiencies reported by the Solids method (Figure 1A) reflect the fact that the mass of solids in a 40 mL sample is relatively small compared to the mass of the liquid. The result aligns with a recent study^58^ which found that assaying the liquid fraction of the influent wastewater led to more sensitive virus detection under typical conditions due to the low solids content of the influent wastewater, despite viruses being more concentrated in the solid fraction. Of all the targets in this study, CV-A6 and Carjivirus were recovered more efficiently by the Solids method (relative to the whole wastewater methods), which could be due to higher partitioning to solids and/or lower recovery by the whole wastewater methods. Interestingly, in a companion paper on wastewater sequencing, we found that Promega and InnovaPrep resulted in a greater reduction in bacteriophage sequences compared to Solids and Nanotrap, which we attributed to the solids removal steps.^59^ Perhaps, a large fraction of the bacteriophage, including Carjivirus, was present inside or attached to the host bacteria and was not effectively released from the solids by the protease (Promega) or surfactant (InnovaPrep) steps prior to solids removal.

### 4.2 Challenges in Conducting and Interpreting Methods Comparison Studies

Some of the recovery efficiencies measured in this study were greater than 100%, which highlights one challenge with spike-in studies: it is not possible to quantify the “true” concentration of target in the spike-in solution. We used the Qiagen AllPrep PowerViral kit to quantify the virus spike-in solution, whereas recovery of the spiked virus targets after mixing with wastewater was performed with the Promega kit, and the recovery appears to have been higher. Recoveries higher than 100% have been reported previously and attributed to the same phenomenon.^19,21^ Our results also illustrate that it is not possible to apply the recovery efficiency measured for a spike-in proxy virus (e.g., bovine coronavirus) to accurately account for the recovery of a target virus because even for the same method, the recovery efficiency varies depending on the target.^21^

Spike-in studies are often used to evaluate the recovery of targets that are not consistently present as endogenous viruses in sufficient concentrations in wastewater. However, lab-cultured viruses added to wastewater may not perfectly mimic the behavior of endogenous viruses, even though we followed methods that have been used by others to allow partitioning of the added viruses to solids.^45^ The fact that we observed patterns of recovery for the two endogenous fecal indicators (Carjivirus and PMMoV) that matched those of the spiked-in viruses lends confidence to our results. Further, variations exist in the procedures of each of the methods we used, which could have an impact on the recovery efficiencies. For example, we did not employ RNA shield or grinding balls in our Solids method,^23^ which could have led to lower recovery efficiencies; it is a limitation of this study that we only included one Solids method. As noted in Discussion [Sec sec4.1.2], some laboratories remove solids via centrifugation before using Nanotrap beads,^60^ which would be expected to reduce the recovery efficiency. The method used for extracting nucleic acids could influence both the recovery efficiency and the inhibition; based on our results, the PowerViral kit produced extracts without significant PCR inhibition.

### 4.3 Implications for Practice

Although we observed differences in the recovery efficiencies across targets and methods, our findings provide encouraging evidence that all four of the methods tested can detect an emerging virus in wastewater. Currently, most surveillance programs are based on observing changes in concentration relative to a baseline value (e.g., CDC NWSS wastewater viral activity level), which reduces the need to account for differences in recovery efficiency between methods, laboratories, and targets. If it is necessary to account for differences in recovery between methods, then we found that normalizing virus concentrations by the concentration of an endogenous fecal indicator (PMMoV or Carjivirus) reduced differences between methods in some cases; however, normalization also increased differences when the pattern of recovery for the indicator was different than the target virus. Thus, it is important to select the appropriate endogenous fecal indicator for this purpose, and there is no single indicator that can account for method-specific differences in recovery for all virus targets and methods.

To enable early detection, it would be ideal to use methods with high sensitivity. While we found that Promega was generally more sensitive than the other methods we tested, it was also more prone to PCR inhibition. The sensitivity of the other methods tested here can be increased by processing larger sample volumes and by pooling results from more than one replicate. It is possible that recovery efficiencies for specific targets could be increased by further optimizing other aspects of the protocols such as extraction kits. Virus stability in wastewater may also affect the suitability of a given method for early detection,^13,14^ which could be important if an emerging virus lyses during its transport through the sewer. Broadening the scope beyond viruses, each of the tested methods has the potential to be applied to other classes of pathogens (e.g., bacteria, protozoan cysts, and fungi); the Promega, InnovaPrep, and Solids methods are not specific to viruses, and the Nanotrap method can be modified by incorporating Nanotrap B particles.^61^ Note also that while our findings should generalize to other types of quantitative PCR, optimal methods to prepare samples for sequencing end points have been shown to be different.^59^ Finally, this study did not address practical aspects of the methods—such as cost, throughput, processing time, etc., and these factors could have significant implications for practice.^19,62^

### 4.4 Future Research Directions

For the application of WBS, little is known about the forms of virus shed by infected individuals,^34,63^ which could impact the persistence of the viral nucleic acid signals in the sewer system^17^ as well as during sample collection and processing. We recommend further research to understand the contribution of extraviral nucleic acids to fecal shedding values. Most studies quantifying recovery efficiencies by concentration and extraction methods do not report the forms of the virus in the samples used, which could vary dramatically for spike-in studies depending on the virus culture and purification methods. We recommend that this information be included in future studies. While we found that the proportions of the dPCR signal due to extraviral nucleic acids varied significantly across the targets we studied, we could not isolate the influence that these differences might have had on the observed recovery efficiencies of the four methods. A more controlled study, in which the proportions of intact virus and extraviral nucleic acids vary over a wide range while keeping other factors constant, would be needed to understand the impacts for each method.

## Supplementary Material





## Notes

Sample metadata, effective volume, and dPCR concentration for spiked-in viruses (XLSX)
Virus stocks acquisition and host cell information; wastewater sample metadata and characteristics; dPCR assay details including primer and probe sequences, dyes, reaction mix, and cycling conditions; dPCR sensitivity results; dMIQE checklist for RT-dPCR experiments; summary of statistical analysis results including recovery efficiency, virus concentrations, and nucleic acid yields figures summarizing the experimental design, virus quantification, partition plots, dPCR inhibition, normalization to fecal indicators, and concentration data in mass-based units; and supplementary methods detailing virus culturing and acquisition, virus form quantification, equations used, and dPCR in-house assay design­(PDF)
