# Supplementary material for: Benchmarking Concentration and Extraction Methods for Wastewater-Based Surveillance of Eight Human Respiratory Viruses: Implications for Rapid Application to Novel Pathogens
Source: Environ Sci Technol. 2025 Aug 29;59(36):19107–18. doi: 10.1021/acs.est.4c13635 (PMC12444993; doi:10.1021/acs.est.4c13635)
Supplement: Supplementary file 2 [file es4c13635_si_002.pdf]

# Supplementary Materials

## Benchmarking concentration and extraction methods for wastewater-based surveillance of eight human respiratory viruses: implications for rapid application to novel pathogens

Audrey Liwen Wang[a], Minxi Jiang[a], Allie Nguyen[a], Staci R. Kane[b], Monica K. Borucki[b], Rose S. Kantor[ab], Kara L. Nelson[a]*

a. Department of Civil and Environmental Engineering, University of California, Berkeley, California 94720, USA
b. Physical and Life Sciences Directorate, Lawrence Livermore National Laboratory, Livermore, California 94550, USA

*Corresponding Author's Email: karanelson@berkeley.edu. Phone: (510) 643-5023

Number of Pages: 24

Number of Tables: 8

Number of Figures: 7

Number of Methods: 5

## Table of Contents

### Supplementary Tables:

**Table S1.** Virus stocks acquisition and host cells information
**Table S2.** Wastewater characteristics (metadata) on each sampling day
**Table S3.** Sample metadata, effective volume, and dPCR concentration (see excel file)
**Table S4.** dPCR forward primer, reverse primer, probe, and dye of every virus target
**Table S5.** dPCR cycling conditions and reaction mix recipe
**Table S6.** dPCR sensitivity LOD/LOQ results
**Table S7.** dMIQE checklist for RT-dPCR experiments
**Table S8.** Statistical analysis of **(A-B)** recovery efficiency for spiked-in viruses, **(C-D)** total gene copies in a 40mL WW sample for endogenous viruses, **(E-F)** purified total nucleic acid concentration, and **(G-H)** total gene copies in a 40mL wastewater sample for spiked-in viruses.

### Supplementary Figures:

**Figure S1.** Overview of the experimental design.
**Figure S2. (A)** Graphical presentation of the calculation of different forms of viruses. **(B)** Concentrations of infectious virus from $TCID_{50}$/mL, intact virus concentration, and total virus concentration
**Figure S3. (A)** Examples of partition fluorescence plots of dPCR positive and negative control. **(B)** Partition fluorescence plots for actual wastewater results of each virus
**Figure S4. (A)** Initial virus concentrations in 40 mL of wastewater based on the concentration of the pure virus cocktail; **(B)** Endogenous virus concentration in 40 mL of wastewater quantified from the unspiked samples; **(C)** Recovery efficiency (%) of the spiked-in viruses across four methods
**Figure S5**. dPCR inhibition for each wastewater source **(A)** EBMUD; **(B)** WCWD; **(C)** SMCSD.
**Figure S6.** Virus concentration reported in mass gram basis across four methods with Solids reported in gc/gTSS and the rest in gc/mLWW
**Figure S7.** Fecal indicators-normalized data

### Supplementary Methods:

**Method A.** Acquisition and culturing of viruses for spiking
**Method B.** Measurement of the forms of virus in the stocks used for spiking
**Method C.** Equations
**Method D.** dPCR in-house assay design
**Method E.** dPCR sensitivity (LOD/LOQ) testing

## Supplementary Tables:

**Table S1.** Virus stocks and host cells information

| Virus family | Strain information | Source and catalog ID | Abbreviation in this manuscript | Host cells |
|---|---|---|---|---|
| Coronaviruses | SARS-Related Coronavirus 2, Isolate USA-WA1/2020, Heat Inactivated | BEI catalog no. NR-52286 | SARS-CoV-2 | N.A |
| | Human coronavirus OC43 strain unknown | BEI catalog no. NR-56241 | OC43 | Vero cells |
| Influenza A viruses | A/California/04/2009 (H1N1)pdm09 | BEI catalog no. NR-13658 | H1N1 | MDCK cells |
| | A/Netherlands/823/1992 (H3N2) | BEI catalog no. NR-49235 | H3N2 | MDCK cells |
| Enteroviruses | Human Coxsackievirus A6 strain Gdula | ATCC VR-1801 | CV-A6 | Rhabdomyosarcoma cells |
| | Human Coxsackievirus B5 strain Faulkner | ATCC VR-185 | CV-B5 | Vero cells |
| Adenoviruses | Human Adenovirus 2 strain unknown | Viral and Rickettsial Diseases Laboratory (VRDL); co-author strain collection | AdV2 | A549 cells |
| | Human Adenovirus 5 strain unknown | | AdV5 | A549 cells |

**Table S2.** Wastewater characteristics (metadata) on each sampling day

| Sampling date | Wastewater site | Flow rate (MGD) | Population | $BOD_5$ (mg/L) | TSS (mg/L) | pH | Per capita flow (L/person/ day) |
|---|---|---|---|---|---|---|---|
| 07/26/2023 | EBMUD | 46.9 | ~700,000 | 330.0 | 320.0 | 7.1 | 253.6 |
| 07/31/2023 | WCWD | 7.3 | ~70,000 | 332.0 | 556.0 | 7.6 | 394.8 |
| 08/02/2023 | SMCSD | 1.3 | ~18,000 | 170.0 | 220.0 | 7.3 | 273.4 |
| 08/30/2023 | EBMUD | 45.9 | ~700,000 | 340.0 | 440.0 | 6.7 | 247.6 |
| 09/05/2023 | WCWD | 7.1 | ~70,000 | 514.0 | 658.0 | 7.6 | 375.4 |
| 09/19/2023 | SMCSD | 1.2 | ~18,000 | 190.0 | 290.0 | 7.6 | 252.4 |

**Table S3:** Sample metadata, effective volume, and dPCR concentration (see excel file)

**Table S4.** dPCR forward primers, reverse primers, probes, and dyes of every virus target. MGB stands for minor groove binder which stabilizes probe-target hybridization and increases melting temperature, allowing the use of shorter probes

| Virus target | Duplex with | Forward primer sequence | Reverse primer sequence | Probe sequence | Dye |
|---|---|---|---|---|---|
| **SARS-CoV-2** | OC43 | GACCCCAAAATCAGCGAAAT | TCTGGTTACTGCCAGTTGAATCTG | ACCCCGCATTACGTTTGGTGGACC | FAM |
| **OC43** | SARS-CoV-2 | TATTGTTCCATGGGTTATGTAC | TCATGCACCTGGTCATAA | /56-TAMN/GG CGG TTT T/ZEN/G GAC ATG TTT ATG ATT T/3IABkFQ/ | TAMRA/ZEN/BkFQ |
| **H1N1** | H3N2 | TTACCAGATTTTGGCRATCTAYT | CCAGGGAGACTASCARTACCA | ACWGTYGCCAGTTC - MGB | FAM/MGB |
| **H3N2** | H1N1 | GCTCAGAGTGGGGAAAGCTATG | TTGGCATAGTCACGTTCAGC | CACGAATCAGATTACAA | VIC/MGB |
| **CV_A6** | CV_B5 | TGACGTGCTGAATGACACAG | AGCCCCTTGGTGAAATTTGC | TGTACCGCTCGGGCTTTTGC | VIC/MGB |
| **CV_B5** | CV_A6 | AAAACTGGCGCACATGAGAC | AACTGGCTCCGTGAATTTCC | AGACTTCACGCAGGACCCGG | FAM/MGB |
| **AdV2** | AdV5 | ACTAAACTTGGAGCGGGT | AGAACACCGTTTTGGTCAA | AAATGATGACAAACTTACCCTGTGGA | FAM |
| **AdV5** | AdV2 | TTGTGCCATCGGTCTACT | GTGGACCAGGTGTTTCAG | GACCTCCCGGCCACTATCC | Texas Red (ROX) |
| **PMMoV** | N.A | GAGTGGTTTGACCTTAACGTTTGA | TTGTCGGTTGCAATGCAAGT | CCTACCGAAGCAAATG | FAM/MGB |
| **Carjivirus** | N.A | CAGAAGTACAAACTCCTAAAAAACGTAGAG | GATGACCAATAAACAAGCCATTAGC | AATAACGATTTACGTGATGTAAC | Texas Red (ROX) |

**Table S5.** dPCR reaction mix recipe and thermal cycling conditions

| DNA virus assays | 8.5k plate | | 26k plate | |
|---|---|---|---|---|
| | singpleplex | duplex | singpleplex | duplex |
| Sample template (ul) | 3 | 3 | 10 | 10 |
| 4x Probe PCR Master Mix (ul) | 3 | 3 | 10 | 10 |
| 10X Primer/probe mix (ul) - virus 1 | 1.2 | 1.2 | 4 | 4 |
| 10X Primer/probe mix (ul) - virus 2 | / | 1.2 | / | 4 |
| Nuclease free H2O (ul) | 4.8 | 3.6 | 16 | 12 |
| Total (ul) | 12 | 12 | 40 | 40 |
| **RNA virus assays** | 8.5k plate | | 26k plate | |
| | singpleplex | duplex | singpleplex | duplex |
| Sample template (ul) | 3 | 3 | 10 | 10 |
| 4x Probe PCR Master Mix (ul) | 3 | 3 | 10 | 10 |
| 100X One-Step RT Mix (ul) | 0.12 | 0.12 | 0.4 | 0.4 |
| 20X Primer/probe mix (ul) - virus 1 | 0.6 | 0.6 | 2 | 2 |
| 20X Primer/probe mix (ul) - virus 2 | / | 0.6 | / | 2 |
| Nuclease free H2O (ul) | 5.28 | 4.68 | 17.6 | 15.6 |
| Total (ul) | 12 | 12 | 40 | 40 |

| # cycles | Temperature °C | Duration |
|---|---|---|
| 1 X | 50 | 40 min |
| 1 X | 95 | 2 min |
| 40 X | 95 | 5 s |
| | 60 | 30 s |

**Table S6:** dPCR sensitivity LOD/LOQ results. See **SI Methods E.** for details on LOD/LOQ determination.

| **8.5K plate (H5N1's HA_FAM assay)** | | | | | | | | | | |
|---|---|---|---|---|---|---|---|---|---|---|
| expected gBlock tube conc. (cps/µL) | 1E+07 | 1E+06 | 1E+05 | 1E+04 | 1E+03 | 1E+02 | 1E+01 | 5E+00 | 6 more samples (2-fold dilutions: 2.5, 1.25,..., 0.078) | 0 (NTC) |
| expected input cps to dPCR (cps/reaction) | 3E+07 | 3E+06 | 3E+05 | 3E+04 | 3E+03 | 3E+02 | 3E+01 | 1.5E+01 | 7.5 - 0.234 | 0 (NTC) |
| measured conc. in dPCR well: average of 3 technical replicates (cps/µL) | - (oversaturated) | 25964.2 | 13633.033 | 1337 | 138.167 | 12.64 | 1.05133 | 1.04067 | 0 < x < 1 | 0 |
| measured conc. In gBlock tube (cps/µL)* | - | 1.04E+05 | 5.45E+04 | 5.35E+03 | 5.53E+02 | 5.06E+01 | 4.21E+00 | 4.16E+00 | - | 0 |
| average positive partitions | 8134.67 | 8256 | 8053.67 | 2857.33 | 359.67 | 34.333 | 3 | 3 | 0 < x < 3 | 0 |
| average valid partitions | 8134.67 | 8257.00 | 8148.33 | 8106.67 | 8087.33 | 8210.67 | 8205.33 | 8164.33 | 8132.56 | 8199.67 |
| average positivity (%) | - (oversaturated) | 99.99% | 98.84% | 35.25% | 4.45% | 0.42% | 0.04% | 0.04% | 0 < x < 0.05% | 0.00% |
| Average CV (CI 95%) | - (oversaturated) | 31.50% | 4.63% | 3.67% | 10.37% | 33.90% | 106.40% | 114.50% | All > 100% | - |
| Example RFU plot for each dilution: | 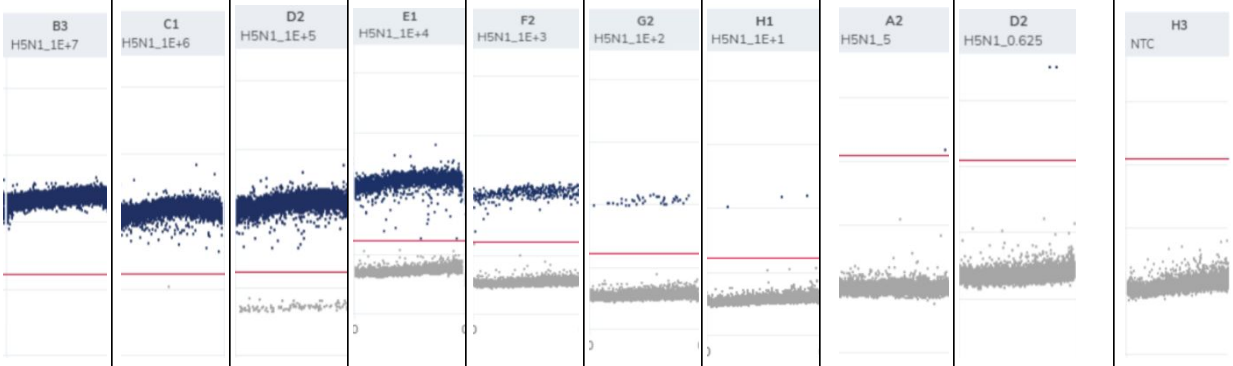 | | | | | | | | | |
| LOD/LOQ | | | These dilutions follow a linear relationship for expected vs. measured gBlock tube conc. (y = 1.0248x - 0.3673, $R^2$ = 0.9997) → LOQ is 100 cps/µL with CV at 33.9% | | | | | | One replicate had no positive partition → LOD = 0.312 cps/µL | |

* This concentration is four times the "measured concentration in the dPCR well (average of 3 technical replicates, cps/µL)," and should be comparable to the "expected gBlock tube concentration (cps/µL)" highlighted in orange.

| 26K plate (H5N1’s HA_FAM assay) | | | | | | | | | | |
|---|---|---|---|---|---|---|---|---|---|---|
| expected gBlock tube conc. (cps/µL) | 1E+05 | 1E+04 | 1E+03 | 1E+02 | 1E+01 | 5E+00 | 2.5E+00 | 1.25E+00 | 5 more samples (2-fold dilutions: 0.625, …, 0.039) | 0 (NTC) |
| expected input cps to dPCR (cps/reaction) | 1E+06 | 1E+05 | 1E+04 | 1E+03 | 1E+02 | 5E+01 | 2.5E+01 | 1.25E+01 | 0.391 - 6.25 | 0 (NTC) |
| measured conc. in dPCR well: average of 3 technical replicates (cps/µL) | - (oversaturated) | 1711.1 | 172.37 | 16.43 | 1.54967 | 0.7733 | 0.45533 | 0.3757 | 0.07 - 0.23 | 0 |
| measured conc. In gBlock tube (cps/µL)* | - | 6.84E+03 | 6.89E+02 | 6.57E+01 | 6.20E+00 | 3.09E+00 | 1.82E+00 | 1.50E+00 | - | 0 |
| average positive partitions | 25436.67 | 18046.33 | 2978.33 | 300.00 | 28.67 | 14.67 | 8.67 | 7.00 | 0.67 - 4 | 0 |
| average valid partitions | 25436.67 | 25473.00 | 25466.67 | 25454.00 | 25474.00 | 25477.67 | 25434.33 | 25475.33 | 25470.00 - 25492.67 | 25467.00 |
| average positivity (%) | - (oversaturated) | 70.84% | 11.70% | 1.18% | 0.11% | 0.06% | 0.03% | 0.03% | 0.01% - 0.03% | 0.00% |
| Average CV (CI 95%) | - (oversaturated) | 1.57% | 3.57% | 11.30% | 37.33% | 51.73% | 68.63% | 75.43% | 99.83% - 107.8% | - |
| Example RFU plot for each dilution: | 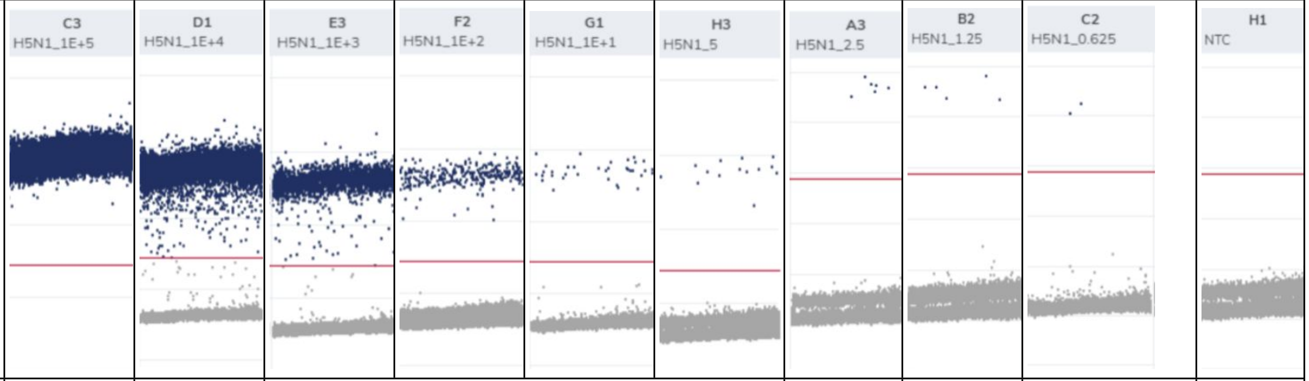 | | | | | | | | | |
| LOD/LOQ | | | These dilutions follow a linear relationship for expected vs. measured gBlock tube conc. (y = 0.9555x - 0.0832, $R^2$ = 0.9914) → LOQ is 5 cps/µL with CV at 51.73% | | | | | | One replicate had no positive partition → LOD = 0.078 cps/µL | |

* This concentration is four times the “measured concentration in the dPCR well (average of 3 technical replicates, cps/µL),” and should be comparable to the “expected gBlock tube concentration (cps/µL)” highlighted in orange.

The limit of detection (LOD)—defined as the concentration at which at least one positive partition is detected in each of the three technical replicates 95% of the time—is 0.312 cps/µL for the 8.5k plate and 0.078 cps/µL for the 26k plate. The limit of quantification (LOQ), defined as the lowest concentration at which results can be reliably and reproducibly quantified, is 100 cps/µL (CV = 33.9%) for the 8.5k plate and 5 cps/µL (CV = 51.73%) for the 26k plate. For the limit of blank (LOB), we included a no-template control (NTC) as a negative control for every assay and plate. All NTCs consistently showed zero positive partitions across all assays and plates.

**Table S7.** Environmental Microbiology Minimum Information (EMMI)[1] Checklist for dPCR

# Environmental Microbiology Minimum Information Checklist

## Study Description

Study: Benchmarking concentration
Date: 22-Sep-2024
Completed by: Audrey Li-Wen Wa

| Environmental Sampling | Sample Treatment | Sample Reduction | Nucleic Acid Extraction | Reverse Transcription | PCR Detection | Analysis |
|---|---|---|---|---|---|---|
| Composite raw wastewater sampling | [x] Performed<br>Protease or Tween20 followed by centrifugation | [x] Performed<br>Concentration by affinity-based bead capture, ultrafiltration, or centrifugation | - Silica column<br>- Heat / mechanical / enzymatic lysis | [x] Performed<br>1-step RT | [ ] qPCR [x] dPCR<br>dPCR | - Threshold adjustment<br>- Data analysis |

## Control Checklist

| | Environmental Sampling | Sample Treatment | Sample Reduction | Nucleic Acid Extraction | Reverse Transcription | PCR Detection |
|---|---|---|---|---|---|---|
| Step performed | [x] | [x] | [x] | [x] | [x] | [x] |
| **Negative Controls** | | | | | | |
| Step has control info | [ ] | [ ] | [ ] | [ ] | [ ] | [x] |
| # control replicates | 0 | 0 | 0 | 0 | 0 | 2 |
| Control result reported | [ ] | [ ] | [ ] | [ ] | [ ] | [x] |
| Data handling reported | [ ] | [ ] | [ ] | [ ] | [ ] | [x] |
| **Positive Controls** | | | | | | |
| Control introduced | [ ] | [ ] | [ ] | [ ] | [ ] | [x] |
| Internal/External | Internal | Internal | Internal | Internal | Internal | External |
| Independent/Parallel | Independent | Independent | Independent | Independent | Independent | Parallel |
| Step has control info | [ ] | [ ] | [ ] | [ ] | [ ] | [x] |
| # control replicates | 0 | 0 | 0 | 0 | 0 | 2 |
| Control result reported | [ ] | [ ] | [ ] | [ ] | [ ] | [x] |
| Data Handling reported | [ ] | [ ] | [ ] | [ ] | [ ] | [x] |

## Process Checklist

### Environmental Sampling
- [x] Sampling Procedure
- [x] Number of samples
- [x] Sample amount, mean, range
- [x] Sampling locations, dates, times

### Sample Treatment
- [x] Performed
- [x] Treatment procedure
- [x] Reagents

### Sample Reduction
- [x] Performed
- [x] Reduction procedure
- [x] Reagents
- [x] Concentration Factor

### Nucleic Acid Extraction
- [x] Extraction procedure
- [x] Amount extracted, amount obtained
- [x] Extract storage conditions

### qPCR or dPCR
- [ ] Target gene name, amplicon length
- [x] Thermocycling temperatures and times
- [x] Master mix: composition, vendors, concentrations
- [ ] Additives: vendors, concentrations
- [x] Template amount added, pre-treatment (if any)
- [x] Primers: sequences, concentrations, vendors, references
- [ ] Amplicon confirmation method (probe, melt curve, etc)
- [x] Probe sequence, concentration, vendor, reference
- [x] Instrumentation
- [x] Equivalent volume of sample analyzed by PCR
- [x] Inhibition assessment procedure
- [ ] Inhibition control description (if used)
- [x] Number samples tested and found inhibited

### Reverse Transcription
- [x] Performed
- [x] One or two step
- [ ] cDNA storage conditions (if two step)
- [x] Reaction temperatures and times
- [x] Reaction reagents and concentrations
- [x] Priming method
- [x] Reaction volume, added template amount
- [x] Inhibition assessment procedure
- [ ] Inhibition control description (if used)
- [x] Number samples tested and found inhibited

### Analysis – dPCR
- [x] Threshold settings
- [ ] Technical replicates, number, well merging
- [x] Partitions measured, number, mean, variance
- [x] Partition volume
- [ ] Target copies per partition, mean, variance
- [x] Program used for dPCR analysis
- [x] Explanation of control results, example plots

### Analysis – qPCR
- [ ] Method for handling failed negative controls
- [ ] Technical replicates, number, calculations
- [ ] Calibration standards: description and source
- [ ] Method of quantifying standards
- [ ] Calibration curve slope
- [ ] Calibration curve R2
- [ ] Lowest standard measured or 95% LOD
- [ ] Cq value determination method

**Table S8:** Statistical analysis of **(A,B)** recovery efficiency for spiked-in viruses; **(C,D)** total gene copies in a 40mL WW sample for endogenous viruses; **(E,F)** purified total nucleic acid concentration, and **(G,H)** total gene copies in a 40mL wastewater sample for spiked-in viruses. (A),(C),(E),(G) p-values of Kruskal-Wallis H-tests that compare differences among concentrations and extraction methods within the same virus type from were shown, with three colors indicating different significance level: yellow is $P < 0.05$, orange is $P < 0.01$, and red is $P < 0.001$ (B),(D),(F),(H) p-values of the Dunn test for pairwise comparisons with Bonferroni correction was further employed following a significant result in the Kruskal-Wallis test. Statistical significance was determined at a 95% confidence interval, with three colors also representing different levels of significance.

**(A)** Kruskal-Wallis test for Figure 1A (recovery efficiency)

| virus | p-value |
|---|---|
| SARS-CoV-2 | 8.20e-04 |
| H1N1 | 2.68e-03 |
| H3N2 | 1.09e-03 |
| OC43 | 8.54e-03 |
| AdV2 | 3.69e-01 |
| AdV5 | 2.82e-02 |
| CV_A6 | 4.37e-01 |
| CV_B5 | 1.04e-03 |

**(B)** Dunn Test for Figure 1A (recovery efficiency)

| AdV5 | Promega | InnovaPrep | Solids | Nanotrap |
|---|---|---|---|---|
| Promega | 1.00e+00 | 1.00e+00 | 2.91e-02 | 1.48e-01 |
| InnovaPrep | 1.00e+00 | 1.00e+00 | 7.85e-01 | 1.00e+00 |
| Solids | 2.91e-02 | 7.85e-01 | 1.00e+00 | 1.00e+00 |
| Nanotrap | 1.48e-01 | 1.00e+00 | 1.00e+00 | 1.00e+00 |

| CV-B5 | Promega | InnovaPrep | Solids | Nanotrap |
|---|---|---|---|---|
| Promega | 1.00e+00 | 1.00e+00 | 1.65e-01 | 1.51e-02 |
| InnovaPrep | 1.00e+00 | 1.00e+00 | 6.82e-02 | 4.89e-03 |
| Solids | 1.65e-01 | 6.82e-02 | 1.00e+00 | 1.00e+00 |
| Nanotrap | 1.51e-02 | 4.89e-03 | 1.00e+00 | 1.00e+00 |

| SARS-CoV-2 | Promega | InnovaPrep | Solids | Nanotrap |
|---|---|---|---|---|
| Promega | 1.00e+00 | 7.66e-02 | 1.22e-03 | 1.00e+00 |
| InnovaPrep | 7.66e-02 | 1.00e+00 | 1.00e+00 | 5.65e-01 |
| Solids | 1.22e-03 | 1.00e+00 | 1.00e+00 | 2.25e-02 |
| Nanotrap | 1.00e+00 | 5.65e-01 | 2.25e-02 | 1.00e+00 |

| OC43 | Promega | InnovaPrep | Solids | Nanotrap |
|---|---|---|---|---|
| Promega | 1.00e+00 | 1.00e+00 | 4.89e-03 | 1.00e+00 |
| InnovaPrep | 1.00e+00 | 1.00e+00 | 1.83e-01 | 1.00e+00 |
| Solids | 4.89e-03 | 1.83e-01 | 1.00e+00 | 1.83e-01 |
| Nanotrap | 1.00e+00 | 1.00e+00 | 1.83e-01 | 1.00e+00 |

| H1N1 | Promega | InnovaPrep | Solids | Nanotrap |
|---|---|---|---|---|
| Promega | 1.00e+00 | 4.28e-02 | 1.75e-03 | 2.79e-01 |
| InnovaPrep | 4.28e-02 | 1.00e+00 | 1.00e+00 | 1.00e+00 |
| Solids | 1.75e-03 | 1.00e+00 | 1.00e+00 | 6.17e-01 |
| Nanotrap | 2.79e-01 | 1.00e+00 | 6.17e-01 | 1.00e+00 |

| H3N2 | Promega | InnovaPrep | Solids | Nanotrap |
|---|---|---|---|---|
| Promega | 1.00e+00 | 2.63e-01 | 3.63e-04 | 2.50e-01 |
| InnovaPrep | 2.63e-01 | 1.00e+00 | 2.76e-01 | 1.00e+00 |
| Solids | 3.63e-04 | 2.76e-01 | 1.00e+00 | 2.90e-01 |
| Nanotrap | 2.50e-01 | 1.00e+00 | 2.90e-01 | 1.00e+00 |

**(C)** Kruskal-Wallis test for Figure 1B (total gene copies in a 40mL WW sample for endogenous viruses)

| virus | p-value |
|---|---|
| PMMoV | 1.43e-02 |
| Carjivirus | 2.80e-02 |

**(D)** Dunn Test for Figure 1B (total gene copies in a 40mL WW sample for endogenous viruses)

| PMMoV | Promega | InnovaPrep | Solids | Nanotrap |
|---|---|---|---|---|
| Promega | 1.00e+00 | 8.14e-02 | 2.28e-02 | 1.00e+00 |
| InnovaPrep | 8.14e-02 | 1.00e+00 | 1.00e+00 | 9.76e-01 |
| Solids | 2.28e-02 | 1.00e+00 | 1.00e+00 | 4.29e-01 |
| Nanotrap | 1.00e+00 | 9.76e-01 | 4.29e-01 | 1.00e+00 |

| Carjivirus | Promega | InnovaPrep | Solids | Nanotrap |
|---|---|---|---|---|
| Promega | 1.00e+00 | 1.00e+00 | 1.00e+00 | 1.83e-01 |
| InnovaPrep | 1.00e+00 | 1.00e+00 | 1.00e+00 | 2.25e-02 |
| Solids | 1.00e+00 | 1.00e+00 | 1.00e+00 | 4.35e-01 |
| Nanotrap | 1.83e-01 | 2.25e-02 | 4.35e-01 | 1.00e+00 |

**(E)** Kruskal-Wallis test for Figure 1C and 1D (conc. in purified TNA for both spiked-in and endogenous viruses)

| virus | p-value |
|---|---|
| AdV2 | 1.03e-01 |
| AdV5 | 1.26e-02 |
| CV_A6 | 9.26e-02 |
| CV_B5 | 7.70e-04 |
| SARS-CoV-2 | 2.09e-04 |
| OC43 | 2.69e-03 |
| H1N1 | 3.92e-03 |
| H3N2 | 3.99e-03 |
| PMMoV | 5.04e-03 |
| Carjivirus | 3.06e-03 |

**(F)** Dunn test for Figure 1C and 1D (conc. in purified TNA for both spiked-in and endogenous viruses)

| AdV5 | Promega | InnovaPrep | Solids | Nanotrap |
|---|---|---|---|---|
| Promega | 1.00e+00 | 1.00e+00 | 7.55e-03 | 1.00e+00 |
| InnovaPrep | 1.00e+00 | 1.00e+00 | 3.00e-01 | 1.00e+00 |
| Solids | 7.55e-03 | 3.00e-01 | 1.00e+00 | 1.83e-01 |
| Nanotrap | 1.00e+00 | 1.00e+00 | 1.83e-01 | 1.00e+00 |

| CV-B5 | Promega | InnovaPrep | Solids | Nanotrap |
|---|---|---|---|---|
| Promega | 1.00e+00 | 1.00e+00 | 1.73e-02 | 1.97e-02 |
| InnovaPrep | 1.00e+00 | 1.00e+00 | 2.56e-02 | 2.91e-02 |
| Solids | 1.73e-02 | 2.56e-02 | 1.00e+00 | 1.00e+00 |
| Nanotrap | 1.97e-02 | 2.91e-02 | 1.00e+00 | 1.00e+00 |

| SARS-CoV-2 | Promega | InnovaPrep | Solids | Nanotrap |
|---|---|---|---|---|
| Promega | 1.00e+00 | 1.96e-02 | 3.73e-04 | 1.00e+00 |
| InnovaPrep | 1.96e-02 | 1.00e+00 | 1.00e+00 | 3.61e-01 |
| Solids | 3.73e-04 | 1.00e+00 | 1.00e+00 | 1.96e-02 |
| Nanotrap | 1.00e+00 | 3.61e-01 | 1.96e-02 | 1.00e+00 |

| OC43 | Promega | InnovaPrep | Solids | Nanotrap |
|---|---|---|---|---|
| Promega | 1.00e+00 | 1.09e-01 | 2.20e-03 | 6.17e-01 |
| InnovaPrep | 1.09e-01 | 1.00e+00 | 1.00e+00 | 1.00e+00 |
| Solids | 2.20e-03 | 1.00e+00 | 1.00e+00 | 3.20e-01 |
| Nanotrap | 6.17e-01 | 1.00e+00 | 3.20e-01 | 1.00e+00 |

| H1N1 | Promega | InnovaPrep | Solids | Nanotrap |
|---|---|---|---|---|
| Promega | 1.00e+00 | 5.19e-02 | 2.64e-03 | 2.44e-01 |
| InnovaPrep | 5.19e-02 | 1.00e+00 | 1.00e+00 | 1.00e+00 |
| Solids | 2.64e-03 | 1.00e+00 | 1.00e+00 | 8.52e-01 |
| Nanotrap | 2.44e-01 | 1.00e+00 | 8.52e-01 | 1.00e+00 |

| H3N2 | Promega | InnovaPrep | Solids | Nanotrap |
|---|---|---|---|---|
| Promega | 1.00e+00 | 4.35e-01 | 1.22e-03 | 1.00e+00 |
| InnovaPrep | 4.35e-01 | 1.00e+00 | 3.30e-01 | 1.00e+00 |
| Solids | 1.22e-03 | 3.30e-01 | 1.00e+00 | 1.07e-01 |
| Nanotrap | 1.00e+00 | 1.00e+00 | 1.07e-01 | 1.00e+00 |

| PMMoV | Promega | InnovaPrep | Solids | Nanotrap |
|---|---|---|---|---|
| Promega | 1.00e+00 | 1.99e-02 | 1.31e-02 | 1.00e+00 |
| InnovaPrep | 1.99e-02 | 1.00e+00 | 1.00e+00 | 6.07e-01 |
| Solids | 1.31e-02 | 1.00e+00 | 1.00e+00 | 4.69e-01 |
| Nanotrap | 1.00e+00 | 6.07e-01 | 4.69e-01 | 1.00e+00 |

| Carjivirus | Promega | InnovaPrep | Solids | Nanotrap |
|---|---|---|---|---|
| Promega | 1.00e+00 | 6.15e-01 | 1.00e+00 | 2.73e-01 |
| InnovaPrep | 6.15e-01 | 1.00e+00 | 1.00e+00 | 1.68e-03 |
| Solids | 1.00e+00 | 1.00e+00 | 1.00e+00 | 6.82e-02 |
| Nanotrap | 2.73e-01 | 1.68e-03 | 6.82e-02 | 1.00e+00 |

**(G)** Kruskal-Wallis test for Figure S4C (total gene copies in a 40mL WW sample for spiked-in viruses)

| virus | p-value |
|---|---|
| AdV2 | 2.12e-01 |
| AdV5 | 5.20e-02 |
| CV_A6 | 4.16e-01 |
| CV_B5 | 8.76e-04 |
| SARS-CoV-2 | 2.09e-04 |
| OC43 | 4.13e-03 |
| H1N1 | 7.86e-03 |
| H3N2 | 8.66e-03 |

**(H)** Dunn test for Figure S4C (total gene copies in a 40mL WW sample for spiked-in viruses)

| CV-B5 | Promega | InnovaPrep | Solids | Nanotrap |
|---|---|---|---|---|
| Promega | 1.00e+00 | 1.00e+00 | 6.82e-02 | 2.56e-02 |
| InnovaPrep | 1.00e+00 | 1.00e+00 | 2.56e-02 | 8.71e-03 |
| Solids | 6.82e-02 | 2.56e-02 | 1.00e+00 | 1.00e+00 |
| Nanotrap | 2.56e-02 | 8.71e-03 | 1.00e+00 | 1.00e+00 |

| SARS-CoV-2 | Promega | InnovaPrep | Solids | Nanotrap |
|---|---|---|---|---|
| Promega | 1.00e+00 | 3.71e-02 | 4.43e-04 | 1.00e+00 |
| InnovaPrep | 3.71e-02 | 1.00e+00 | 1.00e+00 | 6.66e-01 |
| Solids | 4.43e-04 | 1.00e+00 | 1.00e+00 | 2.89e-02 |
| Nanotrap | 1.00e+00 | 6.66e-01 | 2.89e-02 | 1.00e+00 |

| OC43 | Promega | InnovaPrep | Solids | Nanotrap |
|---|---|---|---|---|
| Promega | 1.00e+00 | 9.18e-01 | 1.96e-03 | 1.00e+00 |
| InnovaPrep | 9.18e-01 | 1.00e+00 | 1.83e-01 | 1.00e+00 |
| Solids | 1.96e-03 | 1.83e-01 | 1.00e+00 | 1.48e-01 |
| Nanotrap | 1.00e+00 | 1.00e+00 | 1.48e-01 | 1.00e+00 |

| H1N1 | Promega | InnovaPrep | Solids | Nanotrap |
|---|---|---|---|---|
| Promega | 1.00e+00 | 1.57e-01 | 4.32e-03 | 2.44e-01 |
| InnovaPrep | 1.57e-01 | 1.00e+00 | 1.00e+00 | 1.00e+00 |
| Solids | 4.32e-03 | 1.00e+00 | 1.00e+00 | 1.00e+00 |
| Nanotrap | 2.44e-01 | 1.00e+00 | 1.00e+00 | 1.00e+00 |

| H3N2 | Promega | InnovaPrep | Solids | Nanotrap |
|---|---|---|---|---|
| Promega | 1.00e+00 | 3.20e-01 | 4.17e-03 | 7.33e-01 |
| InnovaPrep | 3.20e-01 | 1.00e+00 | 8.66e-01 | 1.00e+00 |
| Solids | 4.17e-03 | 8.66e-01 | 1.00e+00 | 3.89e-01 |
| Nanotrap | 7.33e-01 | 1.00e+00 | 3.89e-01 | 1.00e+00 |

## Supplementary Figures:

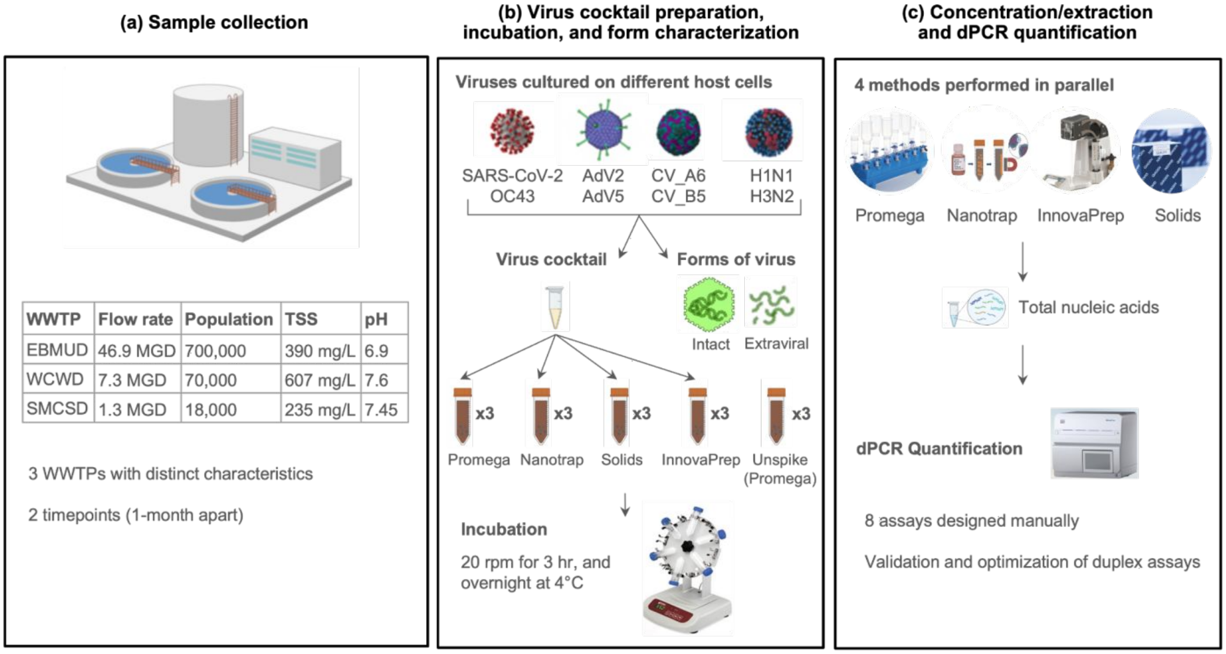


**Figure S1.** Overview of the experimental design. (a) sample collection. (b) virus cocktail preparation, incubation, and form characterization. (c) concentration and/or extraction, and dPCR quantification

**(A)**

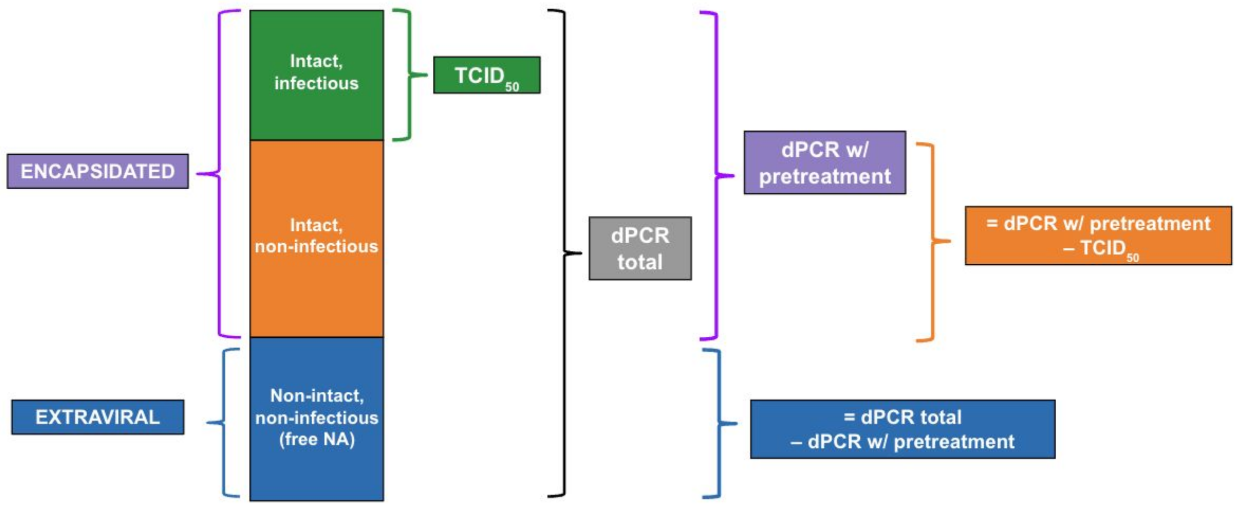


**(B)**

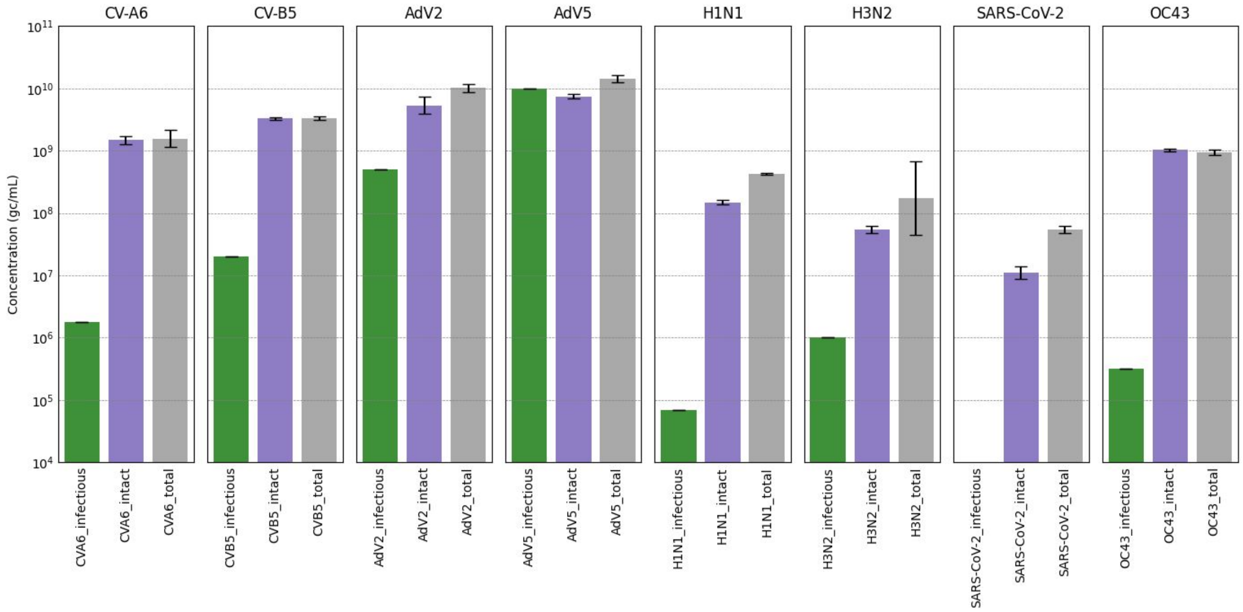


**Figure S2** (**A**) Schematic of the calculation of different forms of viruses. (**B**) Concentrations of infectious virus from $TCID_{50}$/mL (dark green), intact virus concentration (purple) measured after DNAse/RNase treatment pretreatment, and total virus concentration without any treatment (gray). $TCID_{50}$/mL was not determined for SARS-CoV-2, which was purchased as an inactivated stock.

(A)

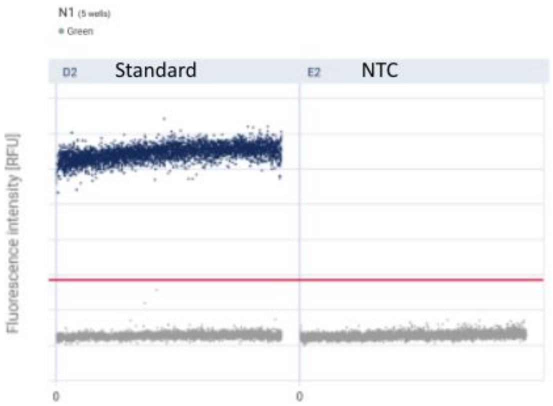


(B)

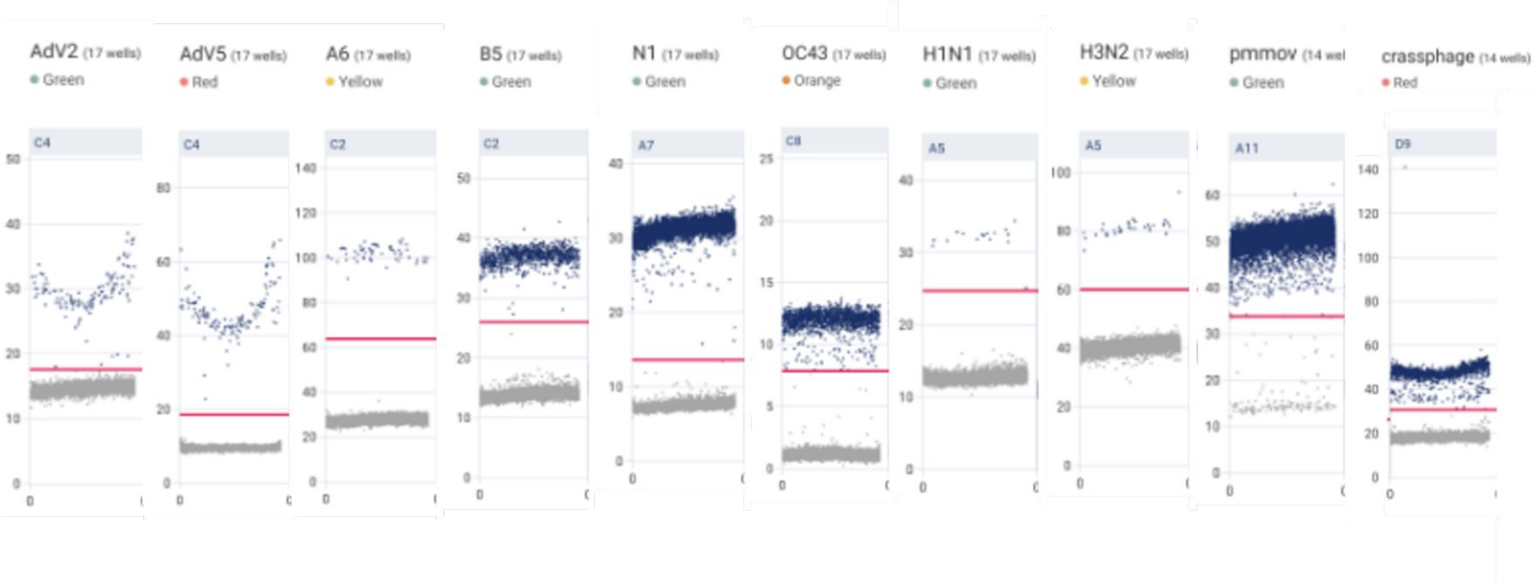


**Figure S3. dPCR partition screenshots (A)** Examples of partition fluorescence plots of dPCR positive and negative control for the CDC N1 assay for SARS-CoV-2 **(B)** Partition fluorescence plots for actual wastewater results of each virus (8 spiked-in viruses and 2 endogenous fecal indicators). Screenshots were adapted from the results of EBMUD time point 1's samples.

**(A)**

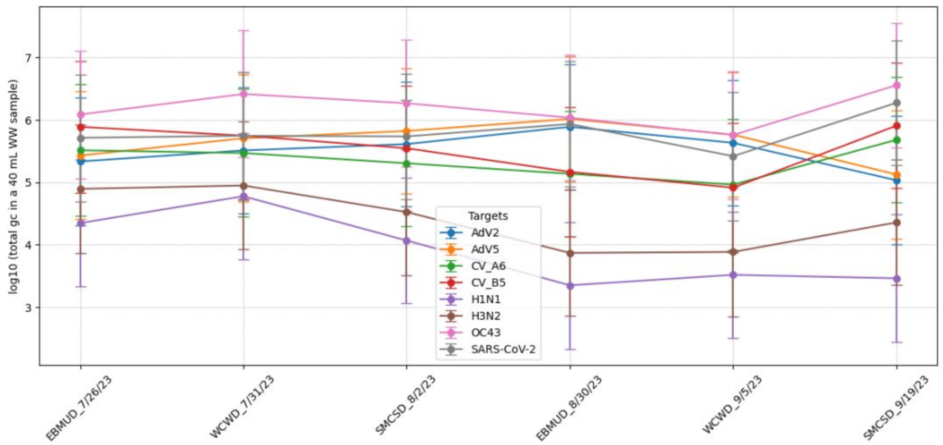
log10 (total gc in a 40 mL WW sample)
Targets
AdV2
AdV5
CV_A6
CV_B5
H1N1
H3N2
OC43
SARS-CoV-2
EBMUD_7/26/23
WCWD_7/31/23
SMCSD_8/2/23
EBMUD_8/30/23
WCWD_9/5/23
SMCSD_9/19/23

**(B)**

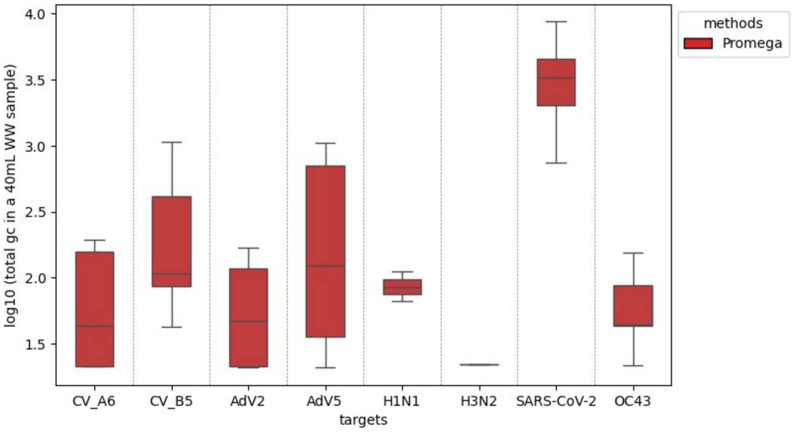
log10 (total gc in a 40mL WW sample)
methods
Promega
CV_A6
CV_B5
AdV2
AdV5
H1N1
H3N2
SARS-CoV-2
OC43
targets

**(C)**

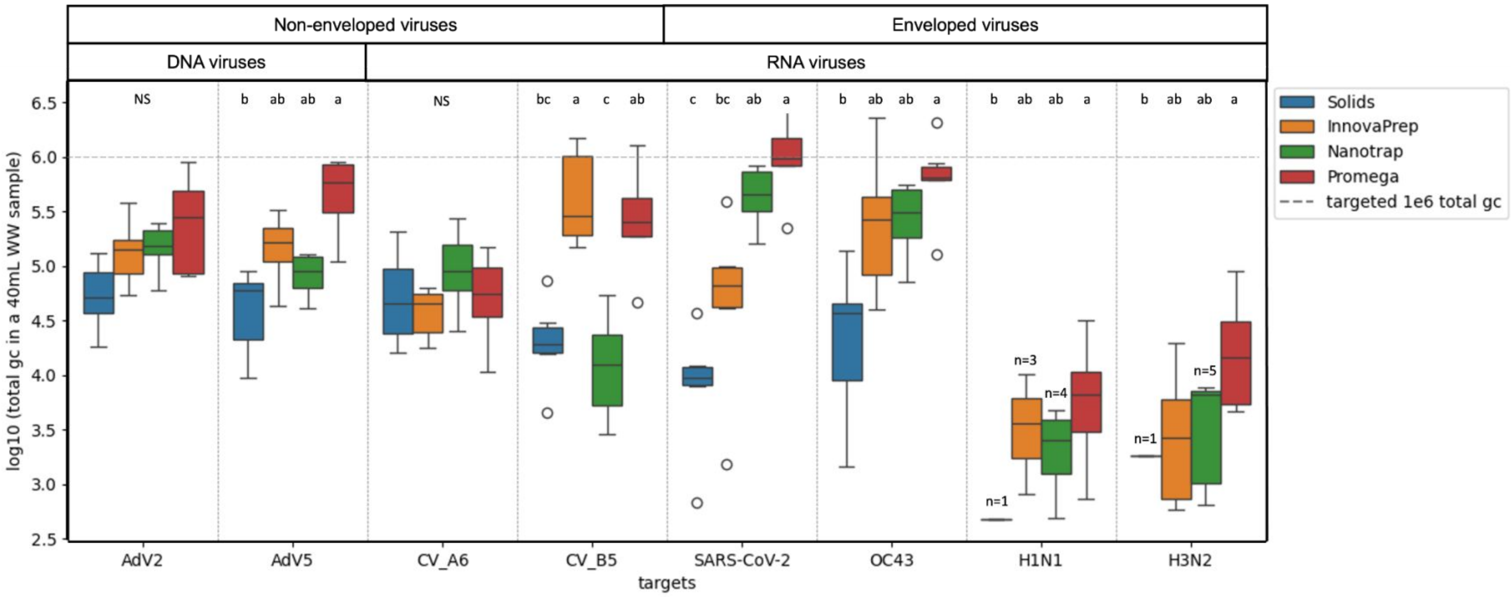
Non-enveloped viruses
Enveloped viruses
DNA viruses
RNA viruses
log10 (total gc in a 40mL WW sample)
Solids
InnovaPrep
Nanotrap
Promega
targeted 1e6 total gc
AdV2
AdV5
CV_A6
CV_B5
SARS-CoV-2
OC43
H1N1
H3N2
targets

**Figure S4.** (**A**) Initial virus gene copies in 40 mL of wastewater based on the concentration of the pure virus cocktail after 4°C refrigeration overnight. The targeted spiked-in gene copies were $10^6$. The pure virus cocktail was quantified after extraction with the Qiagen AllPrep PowerViral extraction kit with carrier RNA addition. Values shown are the geometric mean and standard deviation of triplicate extractions. (**B**) Endogenous virus gene copies in 40 mL of wastewater were quantified from the unspiked samples using Promega for extraction. **(C)** Total gene copies (gc) of spiked-in viruses recovered from a 40 mL of wastewater sample across four concentration / extraction methods (colors); note log10 scale of y-axis. Boxes and whiskers indicate the interquartile range (IQR) and minimum / maximum values within 1.5 times the IQR, respectively, across all samples (n=6 for two time points and three wastewater sources; three biological replicates were averaged first by calculating the geometric mean before plotting). Outliers are displayed as individual points. The initial spike-in was targeted to be $10^6$ gene copies per virus in each wastewater sample (gray dashed line). Significance letters are assigned based on the results of the post-hoc Dunn's test for pairwise comparisons of methods within each virus type. Any method sharing the same letter is not significantly different from each other.

**(A) EBMUD**

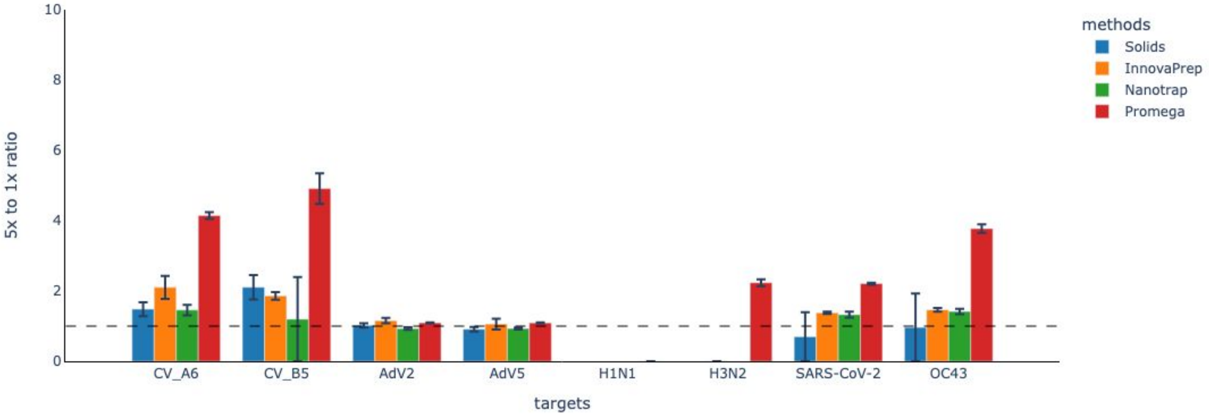


**(B) WCWD**

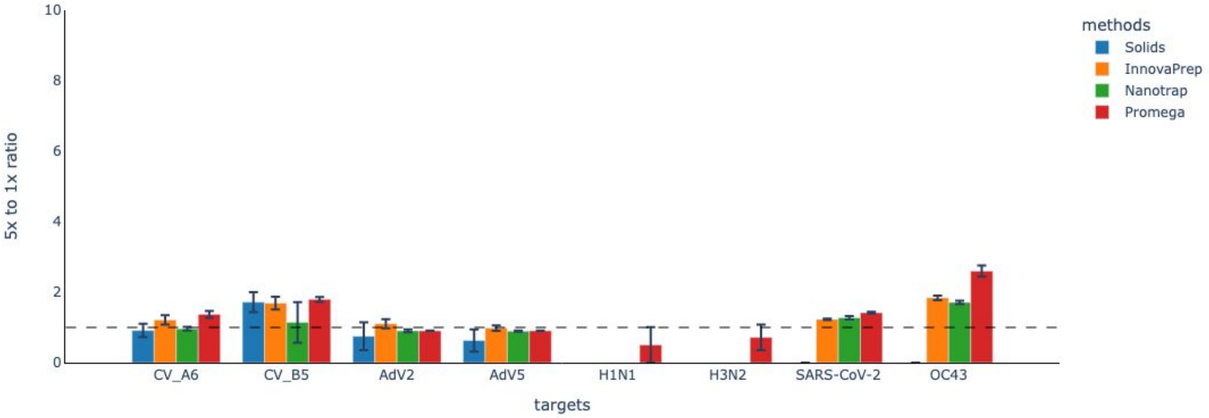


**(C) SMCSD**

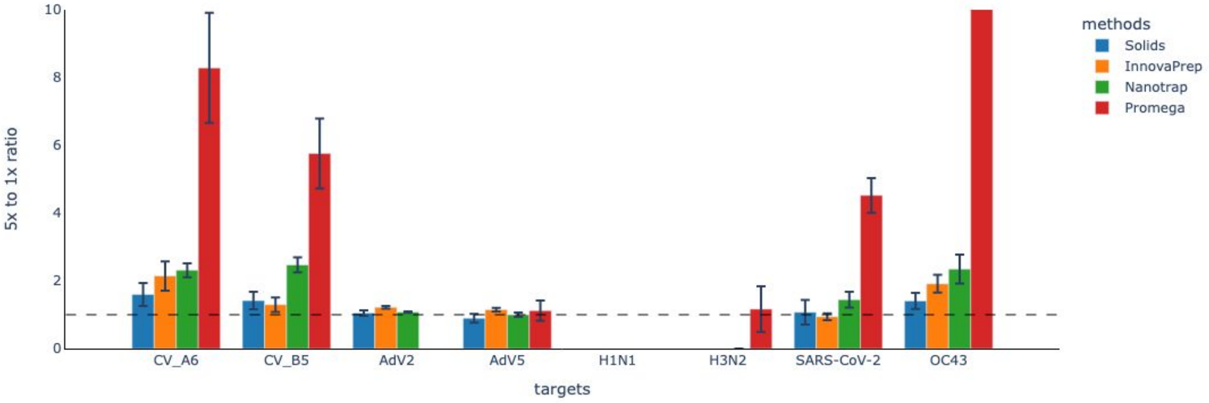


**Figure S5.** dPCR inhibition for each wastewater source **(A)** EBMUD; **(B)** WCWD; **(C)** SMCSD. The purified nucleic acids from each method from time point two were analyzed by dPCR with and without 5-fold dilution. If there are inhibitors present, diluting the sample to 5x reduces the inhibitor concentration, allowing the assay to detect more target TNA. After back-calculating (multiplying the diluted measurement by 5), we should observe a higher concentration if inhibition

was present in the undiluted (1x) sample. Therefore, a ratio of one indicates no inhibition, while higher ratios indicate inhibition. When concentrations were undetectable for either 5x dilution or both 1x and 5x dilutions, the bars are not shown in the figure. Note that the undiluted concentrations of several samples were already low so that the five-fold dilution could result in undetectable signal.

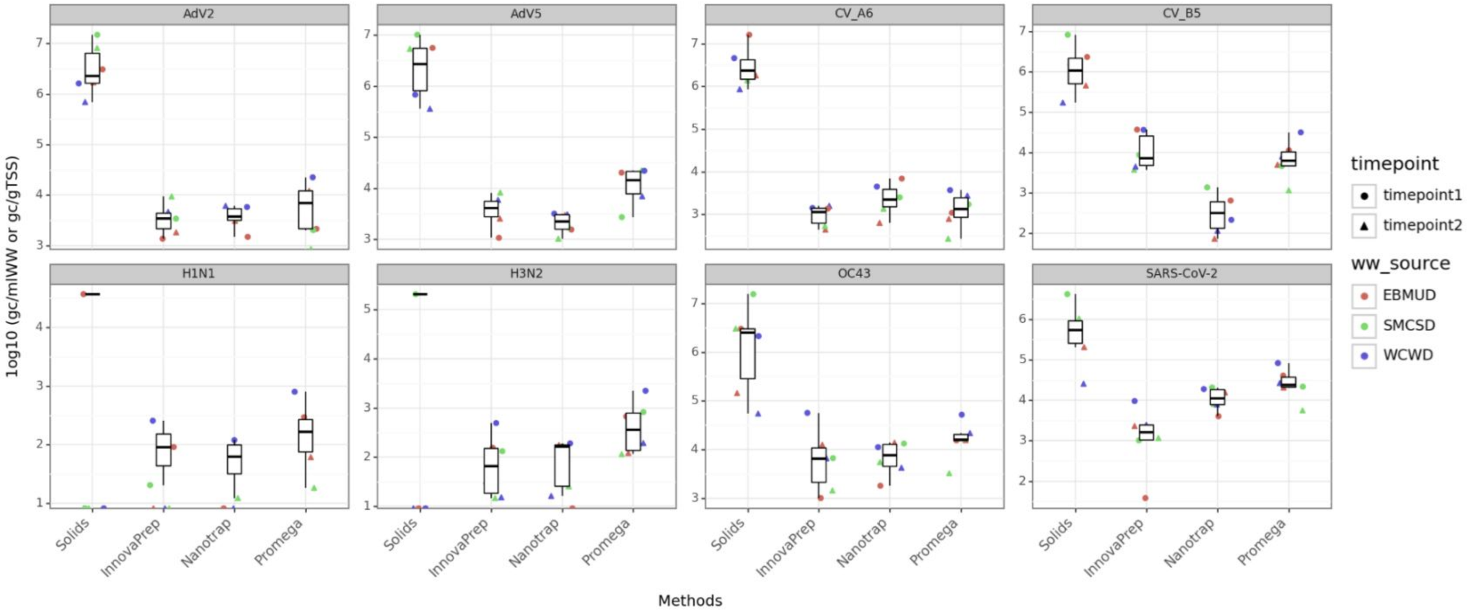


**Figure S6.** Virus concentration reported per mass across four methods with Solids reported in gc/gTSS and the rest in gc/mLWW (note different y-axes for each virus). To calculate the Solids concentration per mass, the TSS concentration (mg/L) provided by the wastewater treatment plant for each sampling date was used. While this mass may not exactly equal that of the solids pellet produced in the laboratory by centrifuging each 40-mL wastewater sample at 20,000 x g, it is likely a good approximation.

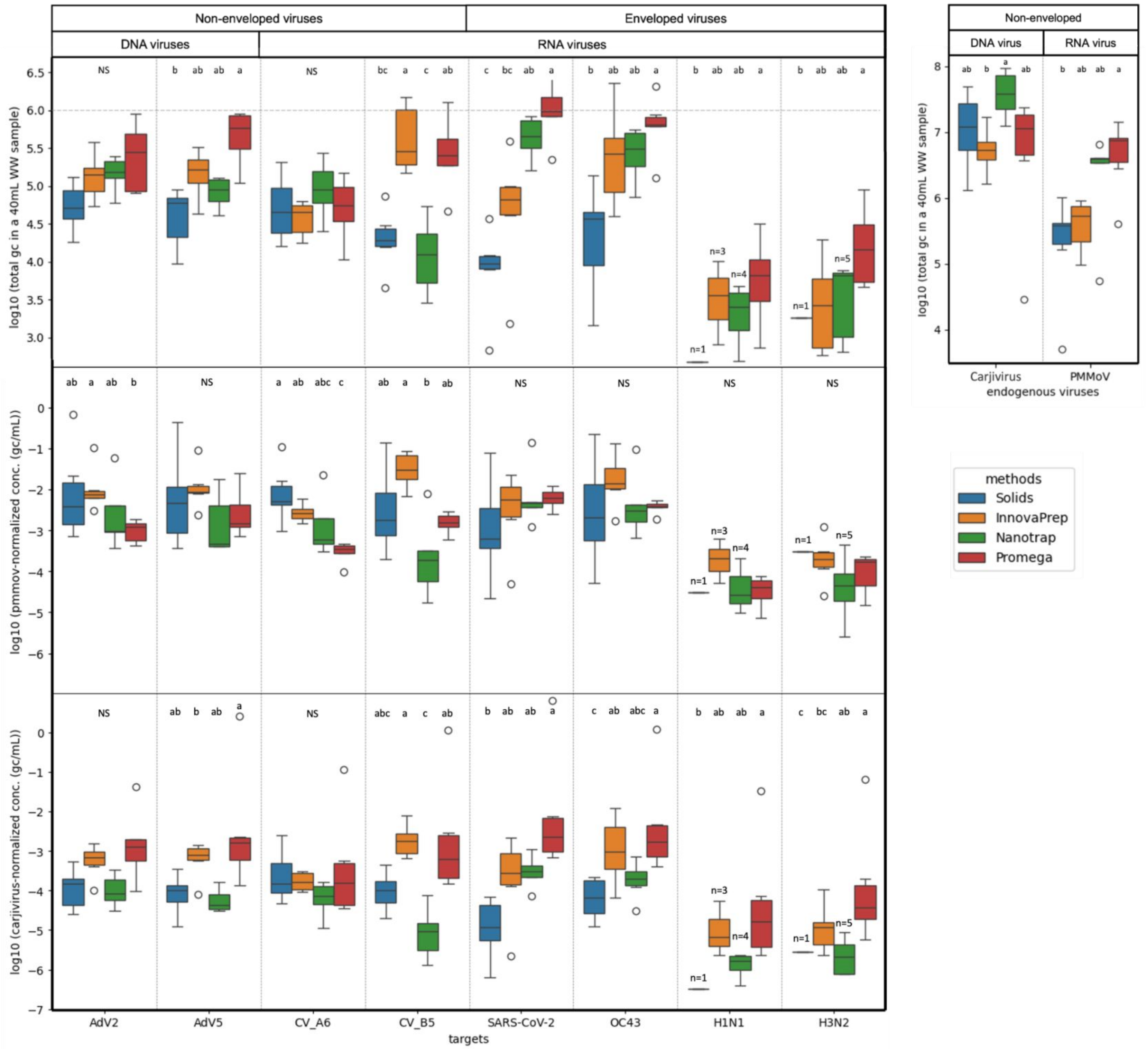


| Virus | Non-normalized p-value | PMMoV-normalized p-value | Carjivirus-normalized p-value |
|---|---|---|---|
| AdV2 | 2.12e-01 | 2.92e-02 | 7.52e-02 |
| AdV5 | 3.03e-03 | 2.24e-01 | 3.06e-03 |
| CV_A6 | 4.16e-01 | 2.85e-03 | 4.73e-01 |
| CV_B5 | 8.76e-04 | 8.11e-03 | 4.81e-04 |
| SARS-CoV-2 | 2.09e-04 | 5.05e-01 | 1.99e-03 |
| OC43 | 4.13e-03 | 2.79e-01 | 3.30e-03 |
| H1N1 | 7.86e-03 | 1.08e-01 | 5.53e-03 |
| H3N2 | 8.66e-03 | 5.00e-02 | 7.23e-04 |

**Figure S7.** Normalized data **(A)** Upper panel is total gc of viruses recovered in a 40-mL WW sample (same as Figure S4C, repeated here to facilitate comparison); the middle panel is PMMoV-normalized data, and the lower panel is Carjivirus-normalized data. **(B)** p-values of Kruskal-Wallis H-tests that compare differences across methods.

## Supplementary Methods

**Method A. Acquisition and culturing of viruses for spiking:**

Adenoviruses Type 2 and 5 were propagated using A549 cells (ATCC CCL-185) at 37°C using Dulbecco's Modified Eagle's Medium (DMEM, Thermo Fisher) with 5% fetal bovine serum (FBS) and penicillin/streptomycin (P/S, 100 U/mL Penicillin & 100 µg/mL Streptomycin; Millipore Sigma). Viral titer was determined via median tissue culture infectious dose ($TCID_{50}$) using A549 cells and calculated according to the Spearman–Kärber method[2] (a more detailed workflow of $TCID_{50}$ is described in the following paragraph). Human coronavirus OC43 (ATCC NR-56241) was propagated on MRC-5 cells (ATCC CCL-171) at 33°C, using DMEM with 5% FBS and P/S according to a previous study[3]. The OC43 titer was determined via $TCID_{50}$ using Vero/TMPRSS2 cells at 37°C using DMEM with 5% FBS and P/S. Coxsackie A6 virus (ATCC VR-1801) was grown on Rhabdomyosarcoma cells (RD; ATCC CCL-136) at 37°C using DMEM with 5% FBS and P/S. The $TCID_{50}$ of CV-A6 was obtained using RD cells. Coxsackie B5 virus (ATCC VR-185) was grown on Vero E6 cells (ATCC CRL-1586) at 37°C using DMEM with 5% FBS and P/S. The $TCID_{50}$ of CV-B5 was obtained using Vero E6 cells. Influenza A virus A/California/04/2009 (H1N1pdm09) and A/Netherlands/823/1992 (H3N2) were grown on MDCK cells (ATCC Cat. No. CCL-34) at 37°C using DMEM with 0.2% BSA, 25 mM HEPES, and 50 U/mL penicillin/50 ug/mL streptomycin (1X). MDCK cells were washed with PBS three times prior to viral infection to remove FBS which would infer with viral production. A concentration of 0.5-1 ug/mL of TPCK-treated trypsin (Sigma Cat. No. T-8642) was added to enhance the infection of influenza viruses in MDCK cells, by cleaving the hemagglutinin precursor protein enabling viral entry. The $TCID_{50}$ of both H1N1 and H3N2 were obtained using MDCK cells.

For all viruses after sufficient time for viral infection of cells (significant cell lysis), the flask contents were removed and centrifuged at 1,000 to 2,800 x g for 5-10 min at 4°C to pellet cell debris. The supernatants were then aliquoted into 1-mL cryotubes and stored at -80C. In addition, coxsackieviruses and adenoviruses were put through freeze/thaw cycles to release virions from cells prior to centrifugation.Finally, heat-inactivated SARS-CoV-2 (isolate: USA-WA1/2020, NR-52286) was obtained from Biodefense and Emerging Infections (BEI) Resources.

To determine $TCID_{50}$ for each virus stock, target cell lines were seeded into 96-well plates and incubated until they reached a monolayer of 95–100% confluency, which was confirmed under a microscope. Prior to infection, virus stocks were serially diluted in 10-fold steps to achieve appropriate ranges for each viral strain. Seven dilutions were used for each virus, with 12 replicate wells per dilution. The last row of each plate served as a no-template control (NTC), in which no virus was added. The dilution ranges were selected based on the expected titer of each virus. For adenoviruses (AdV2 and AdV5) and enteroviruses (CV-A6 and CV-B5), dilutions from $10^{-3}$ to $10^{-10}$ were used. For influenza viruses (H1N1 and H3N2) and coronavirus (OC43), slightly less diluted stocks were used, ranging from $10^{-2}$ to $10^{-9}$. After virus seeding, plates were incubated at 37°C with 5% $CO_2$ and monitored daily for cytopathic effect (CPE). CPE was evaluated under a microscope by comparing infected wells to the NTC controls. CPE phenotypes included cell rounding, swelling, detachment, and patchy areas of cell lysis or clearing. Each well was scored as either positive or negative for CPE based on whether the infected cells showed more degeneration than the control wells. The number of positive and negative wells for each dilution

was recorded, and $TCID_{50}$/mL was calculated using the Spearman-Kärber method, a statistical endpoint dilution analysis. This method estimates the dilution at which 50% of the wells exhibit CPE and provides a measure of infectious virus titer.

**Method B. Measurement of the forms of virus in the stocks used for spiking:**

The DNase pretreatment was performed using a previously established method.[4,5] Enzyme storage buffer consisted of 10 mM Tris-HCl (pH 8.0) and 2 mM $CaCl_2$ in 50% glycerol, and the 10X reaction buffer contained 100 mM Tris-HCl (pH 8.0), 25 mM $MgCl_2$, and 5 mM $CaCl_2$ in Milli-Q water. DNase I, grade II from bovine pancreas (Roche) was resuspended into 5 mL of the storage buffer at a concentration of 40,000 U $mL^{-1}$ and stored at -20 ℃. Immediately before DNase pretreatment of virus stocks, DNase I in storage buffer (40,000 U $mL^{-1}$) was diluted 1:40 in the 10X reaction buffer and gently mixed to obtain a 1,000 U $mL^{-1}$ working DNase solution. The DNase working solution (1000 U/mL) was spiked into virus stock samples to reach a concentration of 200 U/mL and incubated for 30 minutes at 37 $^{o}$C without shaking. Untreated samples were incubated without DNase I at 37$^{o}$C for 30 minutes without shaking. The DNase reaction was inactivated by adding 5 µL of 100 mM EDTA and 5 µL of 100 mM EGTA (per 200 µL of sample) and incubating samples for 5-10 minutes at 65-75 $^{o}$C. The RNase pretreatment was performed according to an established procedure.[5,6] 220 µL of each virus stock were treated with 20 units of RNase ONE Ribonuclease (10 U $\mu L^{-1}$) (Promega). All samples were incubated at 37 $^{o}$C for 15 min with shaking.

**Method C. Equations:**

Virus concentration in purified total nucleic acids ($C_{purified\_TNA}$, gc/µL-TNA) was determined according to **Equation 1**, and the recovered concentration from 40 mL of wastewater ($C_{recovered}$, gc/mL-WW) was calculated based on **Equation 2**.

$$C_{purified\_TNA} = C_{dPCR} (V_{dPCR\_reaction} / V_{dPCR\_template})$$

(**Equation 1**)

Where $C_{dPCR}$ is the concentration reported by dPCR (gc/µL), $V_{dPCR\_reaction}$ is the final reaction volume (µL) for dPCR, $V_{dPCR\_template}$ is the template volume (µL). The volumes were reported in **Table S5**.

$$C_{recovered} = C_{purified\_TNA} (M_{concentrated} / M_{extraction}) (V_{elution} / V_{wastewater})$$

(**Equation 2**)

Where $M_{concentrated}$ refers to the total mass (g) of solid pellets or concentrated liquid (µL) obtained from the Solids method centrifugation or the InnovaPrep concentration step, respectively. The mass was recorded in **Table S3 (column I)**. $M_{extraction}$ represents the actual mass of the solid or liquid sample used for the subsequent extraction step, which is 0.22-0.25 g for the Solids method and 170-200 µL for the InnovaPrep method. $V_{elution}$ is 100 µL, which is the final elution volume from the extraction kit. $V_{wastewater}$ is 40 mL, representing the volume (mL) of one raw wastewater sample.

Recovery efficiency (%) of each virus was calculated as follows:

$$\text{Recovery efficiency (\%)} = [(N_{recovered} - N_{endogenous})/N_{spiked\text{-}in}] \times 100\%$$

(**Equation 3**)

Where $N_{recovered}$ is the number of spiked gene copies recovered by each concentration/extraction method from a 40-mL wastewater sample. $N_{endogenous}$ is the number of endogenous gene copies present in the background of a 40-mL wastewater sample, which was measured using the Promega method. Finally, $N_{spiked\text{-}in}$ is the initial number of gene copies spiked into a 40-mL wastewater sample, which was measured by extracting viruses from the virus cocktail.

**Method D. dPCR virus assay design workflow**

We adopted existing assays from SARS-CoV-2 (CDC N1 assay. 2020) and H1N1pmd09 (WHO Feb 2021). While for other viruses, we designed our in-house assays. We downloaded complete genome sequences for both target and non-target viral groups from NCBI Virus, using taxonomy IDs of the virus candidate as the target and one level above this candidate as non-target. To ensure unique assay design, we masked target genomes using a k-mer-based approach against non-target genomes, eliminating regions with potential cross-reactivity. Using the masked genomes as input, we employed the PriMux tool[7] to generate candidate primer/probe sets. We selected the top-ranked assays based on predicted thermodynamic properties and amplicon length.

Candidate assays were then validated using simulate_PCR[8] against the full NCBI nt database (see the "specificity" column in the table below). We evaluated both sensitivity (coverage of target genomes) and specificity (absence of off-target amplification). We also cross-referenced hit accessions to identify any potential off-targets or missed targets. The final selected assays were ordered from IDT.

In vitro, assays were validated with purified nucleic acids from our cultivated virus strains, IDT gBlocks, and wastewater samples. We confirmed assay performance via standard curves and multiplex compatibility, and included appropriate controls in all dPCR runs. Cross-reactivity and specificity were first assessed in silico by screening against a large database of both target and non-target viral sequences to check for false positives or false negatives. For lab validation, we tested cross-reactivity and specificity by using a mixture of synthetic gBlocks of our target viruses at known concentrations, and no cross-reactivity was observed. When testing the assays in the actual wastewater matrix, we measured background concentrations of all target viruses in the unspiked samples and found them to be significantly lower (Figure S4B) than in the spiked samples (Figure S4C), suggesting that any potential cross-reactivity with untargeted background viruses was minimal or negligible at the spiked concentrations used in this study. The assays would need further testing for specificity and cross-reactivity if the goal is to use them to detect the endogenous wastewater concentrations of these specific viral strains (which was not the use case for our research).

| assay | specificity (via simulate_pcr) | description_of_design | target | antitarget | notes |
|---|---|---|---|---|---|
| Adenovirus 2 | all hAdV2 | masked target against antitarget then ran primux and simulate_pcr | hAdV2 complete genomes | hAdV 1, 5, 6, 57 complete genomes | chose assay based on which one had shortest amplicon, not based on hits |
| Adenovirus 5 | all hAdV5 | masked target against antitarget then ran primux and simulate_pcr | hAdV5 complete genomes | hAdV1, 2, 6, 57 complete genomes | chose assay based on which one had shortest amplicon, not based on hits |
| Human coronavirus OC43 | all OC43 in NCBI | masked target with antitarget (k=15), ran primux and simulate_pcr, identified only 2 primers and 2 probes, chose probe_1 | Human coronavirus OC43 taxid:31631 Nucleotide completeness = complete | Betacoronavirus 1 taxid:694003 Nucleotide completeness = complete (minus OC43) | |
| influenza A virus H1N1pd09 | H1 (includes pd09 and 95% of H1N1 from 2023) | identified in WHO influenza assay source document: WHO Feb 2021 https://cdn.who.int/media/docs/default-source/influenza/molecular-detention-of-influenza-viruses/protocols_influenza_virus_detection_feb_2021.pdf | NA | NA | designed by UK influenza surveillance group |
| influenza A virus H3N2 Texas/1/1977 | H3 just this strain (when compared to the other spike-ins and all 2023 sequences) | made multiple sequence alignment between H3N2 1992 (exact strain), H3N2 1977 (exact strain), and H3N2 2023 (all);<br>identified region with most mismatches between these strains for use as probe;<br>provided chunk of sequence around the probe to primer3plus and had it choose primers (had to use permissive settings)<br><br>simulate_pcr confirmed it hits only this strain | influenza A virus H3N2 Texas/1/1977 | H3N2 from 2023, the H3N2 Netherlands 1992 strain | |
| influenza A virus H3N2 Netherlands/823/1992 | H3 just this strain (when compared to the other spike-ins and all 2023 sequences) | see H3N2 1977 - used same probe and primer positions, modified the sequence to match the 1992 strain | influenza A virus H3N2 Netherlands/823/1992 | H3N2 from 2023, the H3N2 Texas 1977 strain | primux produced assays that primer3 didn't like |
| coxsackievirus B5 ATCC VR 185 | CV-B5, just this strain | masked target against antitarget, then used masked genome of exact strain as input for Primer3Plus | CV-B5 | Enterovirus B taxid: 138949 | primux produced assays that primer3 didn't like |
| coxsackievirus A6 ATCC VR 1801 | CV-A6, just this strain | masked target against antitarget, then used masked genome of exact strain as input for Primer3Plus | CV-A6 | Enterovirus A taxid: 138948 | |

**Methods E. dPCR quality control (QC) and sensitivity (LOD/LOQ) testing**

To pass positive control, the observed dPCR concentrations should align with the expected concentration of the gBlocks; we were expecting a +/- 25% difference in concentration which might be due to pipetting errors or degradation in the freezer. The negative control should have zero positive partitions, resulting in 0 gc/uL in the detected dPCR concentration. Based on the expected and observed gBlock dilutions, the number of valid partitions for accurate quantification was set at 82% (~7000 valid partitions) for 8.5k plates and 76% (~20k valid partitions) for 26k plates. The minimum positivity rate (see Table S6) could be as low as 0.42% for the 8.5k plates, and 0.06% for the 26k plates, which followed a linear relationship between the expected and observed concentrations.

For the sensitivity testing, parameters were recorded according to the guidelines provided by previous studies[1–3]. Concentration range was $10^7$ to $10^1$ in 10-fold dilution, and $10^1$ to 0.0039 in 2-fold dilution. We tested on both the 8.5k and 26k plates, and three technical replicates per dilution were used. We determined LOD/LOQ based on discrete threshold by the mean of three technical replicates. It is important to note that increasing the number of technical replicates generally lowers the estimated LOD/LOQ. The specific criteria for determining LOD is that the probability of positive detection (at least one positive partition) should be 95% (meaning all three wells should have at least one positive partition). For LOQ precision, we set the CV (%) threshold based on if the concentration follows a linear relationship with expected dilutions. Based on the LOQ testing across multiple dilutions in our study, we found that the 26k plate consistently reported concentrations that were on average 21.5% higher than those measured by the 8.5k plate. Based on the 8.5k plate results, the dilutions ranging from $10^2$ to $10^5$ cps/μL (corresponding to an average of 34.33 to 8053.67 positive partitions) follow a linear relationship ($y = 1.0248x - 0.3673$, R2 = 0.9997), concentrations within this range is expected to have an accurate quantification.

## References:


1. Borchardt MA, Boehm AB, Salit M, Spencer SK, Wigginton KR, Noble RT. The Environmental Microbiology Minimum Information (EMMI) Guidelines: qPCR and dPCR Quality and Reporting for Environmental Microbiology. *Environ Sci Technol*. 2021;55(15):10210-10223. doi:10.1021/acs.est.1c01767

2. Lei C, Yang J, Hu J, Sun X. On the Calculation of TCID50 for Quantitation of Virus Infectivity. *Virol Sin*. 2020;36(1):141-144. doi:10.1007/s12250-020-00230-5

3. Savoie C, Lippé R. Optimizing human coronavirus OC43 growth and titration. *PeerJ*. 2022;10:e13721. doi:10.7717/peerj.13721

4. Thornton JE. DNase I Treatment. Published online August 28, 2015. Accessed November 14, 2024. https://protocols.io/view/DNase-I-Treatment-c3myk5

5. Harrison KR, Snead D, Kilts A, Ammerman ML, Wigginton KR. The Protective Effect of Virus Capsids on RNA and DNA Virus Genomes in Wastewater. *Environ Sci Technol*. 2023;57(37):13757-13766. doi:10.1021/acs.est.3c03814

6. Rockey N, Young S, Kohn T, et al. UV Disinfection of Human Norovirus: Evaluating Infectivity Using a Genome-Wide PCR-Based Approach. *Environ Sci Technol*. 2020;54(5):2851-2858. doi:10.1021/acs.est.9b05747

7. PriMux. SourceForge. December 30, 2015. Accessed November 14, 2024. https://sourceforge.net/projects/primux/

8. Gardner SN, Slezak T. Simulate_PCR for amplicon prediction and annotation from multiplex, degenerate primers and probes. *BMC Bioinformatics*. 2014;15(1):237. doi:10.1186/1471-2105-15-237
